# Identification of genes and mutations involved in antimicrobial low susceptibility in *Mycoplasma bovirhinis* isolates from Japan and development of a genetic method for antimicrobial susceptibility discrimination

**DOI:** 10.1128/spectrum.00607-26

**Published:** 2026-06-12

**Authors:** Eiji Hata

**Affiliations:** 1 Arthropod-borne Disease Group, Department of Transboundary Animal Diseases, National Institute of Animal Health (NIAH), National Agriculture and Food Research Organization (NARO), Kagoshima, Kagoshima, Japan; Michigan State University, East Lansing, Michigan, USA

**Keywords:** *Mycoplasma bovirhinis*, antibiotic resistance, prophage, genetic determination of antimicrobial susceptibility

## Abstract

*Mycoplasma bovirhinis* (*Mycoplasmopsis bovirhinis*) is a commensal bacterium that infects the bovine respiratory tract and poses a risk for calf pneumonia. This study aimed to develop a simple and rapid genetic method for assessing antimicrobial susceptibility by identifying genes and mutations associated with decreased susceptibility through genomic analysis of isolate exhibiting low antimicrobial susceptibility and by directly detecting these genetic determinants. As a result, phage-related regions encoding homologous genes—namely, aminoglycoside 6-adenylyltransferase, aminoglycoside 3′-phosphotransferase, and a tetracycline ribosomal protection protein—associated with decreased susceptibility to aminoglycosides or tetracyclines were identified. Furthermore, point mutations associated with decreased susceptibility to macrolides, lincomycins, and fluoroquinolones were identified in the 23S ribosomal RNA gene (*rrl*), DNA gyrase gene (*gyrA*), DNA topoisomerase IV gene (*parC*), and its homologous gene (*parC*′). Field strains harboring these determinants and mutations have been widely detected in Japan since 2001. These genes and mutations can be easily and rapidly detected by PCR, DNA sequencing, and melting curve analysis using hybridization probes, making it possible to identify effective antimicrobial agents prior to antimicrobial treatment, which was not possible with conventional, time-consuming, culture-based antimicrobial susceptibility testing for mycoplasmas. This approach is expected to improve cure rates and reduce the risk of using ineffective antibiotics.

**IMPORTANCE:**

Antimicrobial susceptibility testing for mycoplasma using conventional culture methods takes a long time due to the slow culture rate. This has been a major problem in selecting effective antibacterial agents for antimicrobial therapy. Therefore, we identified the genes and mutations involved in decreased susceptibility and developed a genetic susceptibility determination method that can directly detect them. This method makes it possible to quickly and easily detect the antimicrobial susceptibility characteristics of mycoplasmas, which have been difficult to understand until now. In recent years, the emergence and spread of low-susceptibility field strains of various bovine mycoplasmas has been confirmed, raising concerns about this situation. Utilizing this method is expected to improve cure rates and reduce the use of antimicrobial agents.

## INTRODUCTION

*Mycoplasma bovirhinis* (*Mycoplasmopsis bovirhinis*) is a commensal bacterium that inhabits the bovine respiratory and reproductive tracts and can become a secondary cause of calf pneumonia complex (1, 2). However, many aspects of antibiotic treatment targeting this bacterium remain unclear, including its effects on the pathogenesis of calf pneumonia, and clear guidelines for antibiotic therapy have not yet been established in Japan. Mycoplasmal calf pneumonia has been increasing in prevalence throughout the world in association with the dissemination of large-scale livestock management and high stocking density, and the economic impact caused by this condition is considerable (3).

In recent years, the emergence of field isolates with low antimicrobial susceptibility has been reported for various bovine *Mycoplasma* species (i.e., *M. alkalescens*, *M. bovirhinis*, *M. bovis*, and *M. californicum*) (4–7). Tetracyclines, macrolides, lincosamides, fluoroquinolones, phenicols, pleuromutilines, and some aminoglycosides are recognized as the primary antimicrobial agents for the treatment of mycoplasmal infections in animals (8). Depending on the bovine *Mycoplasma* species, most decreases in susceptibility to these antimicrobial agents have been associated with mutations in genes encoding target factors (e.g., ribosome, ribosomal proteins, DNA gyrase, and DNA topoisomerase IV) (5–9). Although not the primary mechanism, decreased antimicrobial susceptibility due to the acquisition of efflux pump activity or modification activity has also been reported for *M. bovirhinis*, *M. hominis*, and *M. pneumoniae* (10–12). Furthermore, a correlation has been reported between biofilm-forming ability and decreased antibiotic susceptibility in *M. gallisepticum*, *M. synoviae*, and *M. hyopneumoniae* (13).

Identifying genes and mutations that confer decreased susceptibility to antimicrobial agents should lead to the development of genetic methods for determining antimicrobial susceptibility. In the case of *Mycoplasma*, it is difficult to easily and quickly determine antimicrobial susceptibility using conventional culture-based antimicrobial susceptibility tests because slow growth is a bacterial characteristic of *Mycoplasma,* and colony formation usually takes 2 or more days. No simple method for determining antimicrobial susceptibility equivalent to the Kirby‒Bauer test or E test has been developed (13, 14). Conventional culture-based antimicrobial susceptibility tests, such as the agar plate dilution method and the broth microdilution method, require 2 to 3 weeks for results to be determined, making it difficult to reflect the results of antimicrobial susceptibility testing in treatment (13, 14). By contrast, genetic antimicrobial susceptibility testing methods (based on PCR, DNA sequencing, and melting curve analysis) can directly detect genes and mutations involved in decreased susceptibility, making test results easier and clearer to interpret (5–7, 13). Other advantages of these methods are their simple testing procedure and low cost (5, 6, 13). The fact that the genetic testing-based method does not require culture medium components, antibacterial agents, or quality control strains required in conventional methods is important for its widespread use. Other unresolved issues with conventional methods for veterinary mycoplasmas include the lack of standardized testing methods and the absence of established clinical breakpoints and epidemiological cutoff values (15). Identifying effective antimicrobial agents prior to antimicrobial treatment is important for both increased treatment effectiveness and decreased use of less-effective antimicrobial agents.

In this study, we aimed to clarify the actual antimicrobial susceptibility status of *M. bovirhinis* in Japan and to identify genes and mutations involved in reduced susceptibility. We also aimed to establish genetic antimicrobial susceptibility testing targeting these genes and mutations.

## RESULTS

### Antimicrobial susceptibility testing

**Fig 1:**
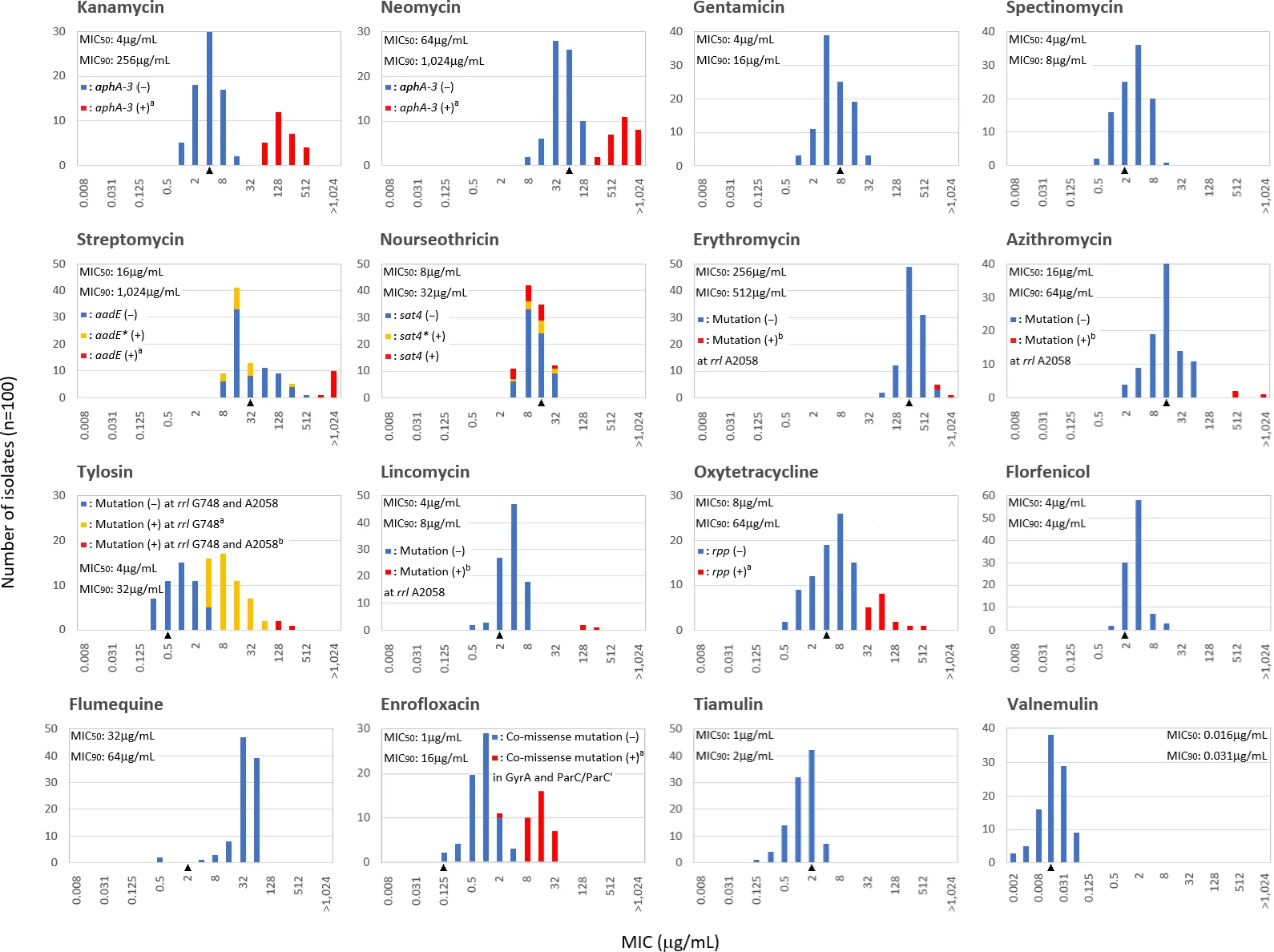
Relationship between antimicrobial susceptibility distributions and the presence of genes and mutations associated with decreased antimicrobial susceptibility. MIC values for strain PG43^T^ are indicated by a black triangle beneath the bar graph. *M. bovirhinis* typically harbors *rrl* A2057 and is unable to form a base pair with *rrl* C2611, thereby exhibiting intrinsic resistance to erythromycin. Significant differences in MICs associated with the presence of each gene or mutation were evaluated by the Mann‒Whitney *U* test. ^a^*P* < 0.0001; ^b^*P* < 0.01. No mutations associated with decreased susceptibility to tetracycline (*rrs* A965 and *rrs* A967) or spectinomycin (*rrs* C1192), as reported in other Mycoplasma species, were identified in the *M. bovirhinis* field isolates. Meaning of abbreviations: *aphA-3*, aminoglycoside 3′-phosphotransferase APH(3′)-IIIa gene; *aadE*, aminoglycoside 6-adenylyltransferase ANT (6) gene; *sat4*, GNAT family N-acetyltransferase gene; *rrl*, 23S ribosomal RNA gene; *rpp*, tetracycline ribosomal protection protein gene. Genes whose functions are presumed to be impaired due to replacement are marked with an asterisk (*).

Figure 1 shows the minimum inhibitory concentration (MIC) distributions of the 16 antimicrobial agents tested against *M. bovirhinis* field isolates, the MIC_50_ and MIC_90_ values representing the antimicrobial concentrations that inhibited the growth of 50% and 90% of the test strains, respectively, and the MICs for strain PG43^T^ (MIC_PG43_^_T_^ [a quality control reference isolate]). The antimicrobial agent to which the isolates exhibited the highest susceptibility was valnemulin, with MIC_50_ and MIC_90_ values of 0.016 µg/mL and 0.031 µg/mL. The MICs of gentamicin, spectinomycin, nourseothricin, flumequine, florfenicol, tiamulin, and valnemulin exhibited normal distributions, and the MIC for strain PG43^T^ also fell within each MIC range, so all *M. bovirhinis* isolates were considered to remain susceptible to these antimicrobial agents. On the other hand, the MIC distributions of several antimicrobial agents (i.e., kanamycin, neomycin, streptomycin, tylosin, oxytetracycline, and enrofloxacin) are clearly bimodal. Moreover, a total of three isolates with low susceptibility to azithromycin and lincomycin were sporadically detected.

Many bovine mycoplasmal species show natural resistance to 14-membered macrolides and older quinolones (8, 13). Indeed, *M. bovirhinis* field isolates and PG43^T^ showed extremely high MIC values for erythromycin (MIC_50_: 256 µg/mL, MIC_90_: 512 µg/mL, MIC_PG43_^_T_^: 256 µg/mL), *M. bovirhinis* field isolates showed high MIC values for flumequine (MIC_50_: 32 µg/mL, MIC_90_: 64 µg/mL).

### Genome-based identification of candidate factors that decrease antimicrobial susceptibility in HAZ2616

HAZ2616 is an isolate that shows low susceptibility to kanamycin, neomycin, streptomycin, and oxytetracycline. The MIC values of these agents are 256 µg/mL, >1,024 µg/mL, >1,024 µg/mL, and 512 µg/mL, respectively. The total genome size of HAZ2616 is 947,864 bp, with 56.5 × coverage; the guanine‒cytosine (GC) content is 28.09%, and the genome sequence is registered under GenBank accession no. AP038809.

The deduced amino acid (aa) sequences of 66 of the 71 coding sequences (CDSs) located between locus_tag MBVR2616_0484 and 0555 show high similarity to the deduced aa sequences of genes from bacteria other than mycoplasmas. This DNA region was 58,624 bp in length with a GC content of 36.06%. Similar to the prophage-like region of HAZ141_2 (GenBank accession no. AP018135), this DNA region significantly exceeded the average GC content (28%‒29%) of mycoplasmal genomes, including that of *M. bovirhinis* HAZ2616 (12). In *Mycoplasma* species, TGA functions as a tryptophan (Trp) codon rather than a stop codon, which can result in elongation of the polypeptide chain in foreign genes. This phenomenon was observed by multiple genes in the prophage-like region. (e.g., locus_tags MBVR2616_0498, 0501, 0504, 0513, 0520, 0545).

**Fig 2:**
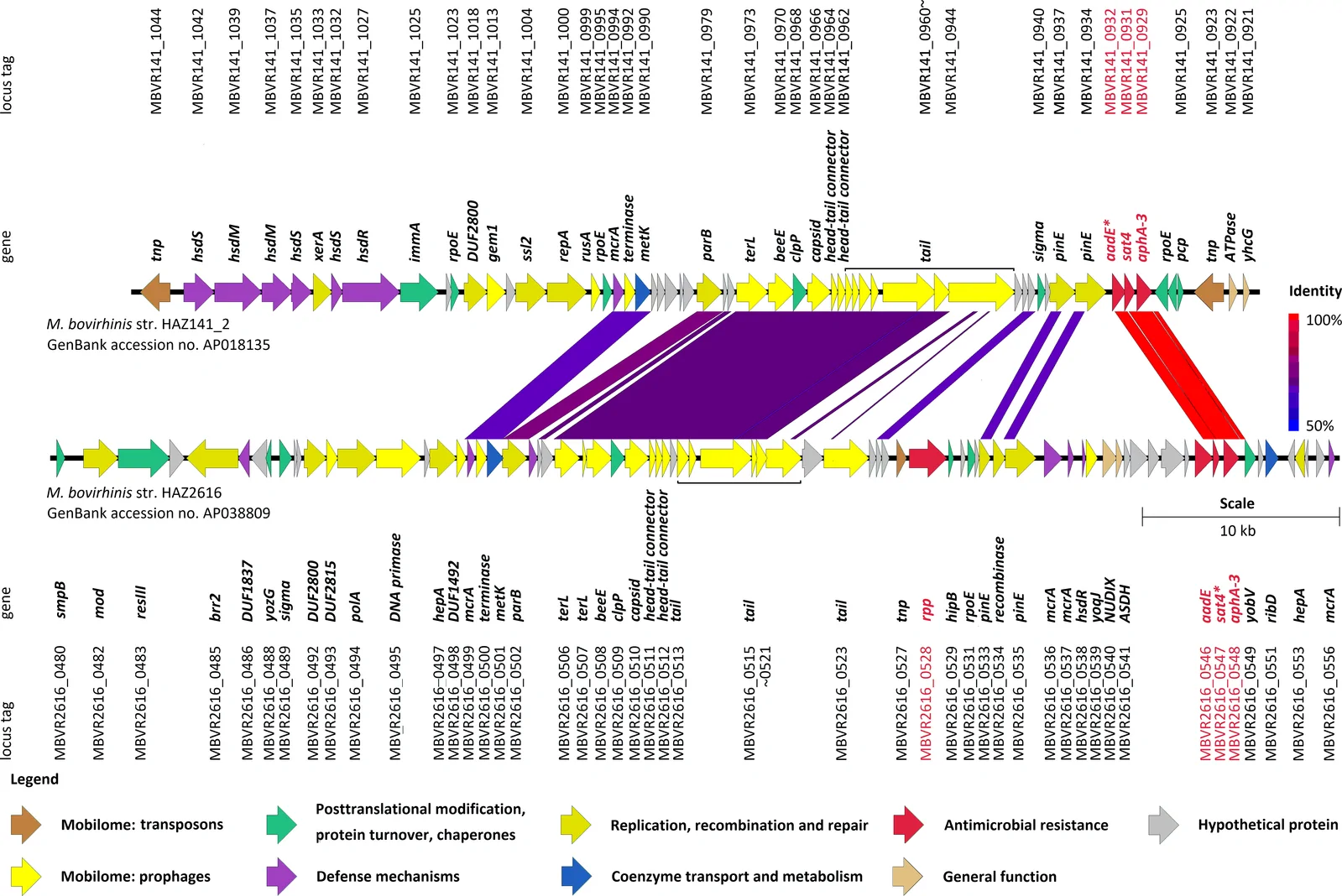
Comparison of a prophage-like genomic region between *M. bovirhinis* HAZ141_2 (GenBank accession no. AP018135) and *M. bovirhinis* HAZ2616 (GenBank accession no. AP038809). Arrows represent open reading frames (ORFs), with the arrowheads indicating the direction of transcription. The ORFs are color-coded based on the putative functions of the predicted proteins. Amino acid sequence identities were analyzed using Easyfig (16), and the percentage identity is indicated by a color gradient (red to blue). Genes and locus tags associated with decreased antimicrobial susceptibility are highlighted in red.

Moreover, many CDSs related to phage components are located between locus_tags MBVR2616_0493 and 0523, and the aa sequences and the order of these CDSs show high similarity to the prophage-like region of HAZ141_2 (Fig. 2). These genomic characteristics suggest that this DNA region in HAZ2616 originates from other bacterial genomes and is highly related to the prophage-like region of HAZ141_2.

In bacteria other than mycoplasmas, the acquisition of factors involved in antimicrobial inactivation (e.g., antimicrobial-modifying enzymes) is considered a major mechanism of decreased susceptibility, but this is rare in mycoplasmas (8). However, in the genome sequence of *M. bovirhinis* strain HAZ141_2, a gene cluster encoding homologs of aminoglycoside-modifying enzymes (aadE*-sat4-aphA-3) was identified in a genetic region that was putatively inserted from other bacterial species (12, 17). These are homologs of aminoglycoside 6-adenylyltransferase ANT(6) (*aadE*), GNAT family N-acetyltransferase (*sat4*), and aminoglycoside 3′-phosphotransferase APH(3′)-IIIa (*aphA-3*). These genes are also present in the genome sequence of HAZ2616 (Fig. 2). The cluster of three aminoglycoside-modifying genes (aadE-sat4*-aphA-3) is located downstream of the 3′-terminus to the CDSs associated with phage components, as observed in the prophage-like region in HAZ141_2. Although 70 bp of overlap between aadE* and *sat4* was reported in the prophage-like region of HAZ141_2 (12), *aadE* and sat4* also overlap by 16 bp in this DNA region in HAZ2616. Moreover, a gene encoding an aa sequence similar to the tetracycline ribosomal protection protein (Rpp) reported in various bacteria was identified in the genome sequence of HAZ2616, and *rpp* is located between the CDSs associated with phage components and aadE-sat4*-aphA-3 (Fig. 2).

Of 100 field isolates, there were 17 aadE*-sat4-aphA-3-carrying isolates, 11 aadE-sat4*-aphA-3-carrying isolates, and 17 *rpp*-carrying isolates, and five isolates that simultaneously carried aadE-sat4*-aphA-3 and *rpp*.

These phage-related DNA regions confirmed in HAZ141_2 and HAZ2616 appear to be widely distributed among Japanese field strains as genetic factors that confer low susceptibility to certain antimicrobial agents.

In *M. bovis*, the quinolone resistance-determining regions (QRDRs) are located in the DNA gyrase A subunit (GyrA) and the DNA topoisomerase IV C subunit (ParC) (6). The deduced aa sequences of ParC (locus_tag NCTC10118_00431, 833 aa, ParC_PG43^T^; locus_tag MBVR141_0287, 851 aa, ParC_HAZ141_2; locus_tag CO229_00195, 851 aa, ParC_GS01) from the three *M. bovirhinis* strains whose genome sequences have been determined (i.e., PG43^T^ [GenBank accession no. LR214972], HAZ141_2, and GS01 [GenBank accession no. CP024049]) are highly similar to one another, with over 95.4% (812 aa/851 aa) aa sequence identity. On the other hand, the ParC (locus_tag MBVR2616_0044, 853 aa, ParC′_HAZ2616) from HAZ2616 is less than 75.9% (647 aa/853 aa) identical to the ParC from PG43^T^, HAZ141_2, and GS01, suggesting that this protein represents a distinct homolog of the previously recognized ParC of *M. bovirhinis*. Therefore, the ParC that shows ≥95% aa sequence identity with ParC from HAZ2616 was referred to as ParC′ (Fig. S1A). Of the field isolates, *parC* only, *parC*′ only, and the coexistence of *parC* and *parC*′ were detected in 26, 3, and 71 isolates, respectively, revealing that not only *parC* but also *parC*′ coexist in many field isolates. Incidentally, the aa sequence of GyrA deduced from HAZ2616 (locus_tag MBVR2616_0560, 870 aa, GyrA_HAZ2616) is nearly identical to GyrA from PG43^T^, HAZ141_2, and GS01 (locus_tag NCTC10118_00196, 870 aa, GyrA_PG43^T^; locus_tag MBVR141_0545, 868 aa, GyrA_HAZ141_2; locus_tag CO229_01205, 870 aa, GyrA_GS01), with an identity of over 98.7% (859 aa/870 aa) (Fig. S1B).

### Identifying genes and mutations associated with decreased susceptibility to antimicrobial agents

**Fig 3:**
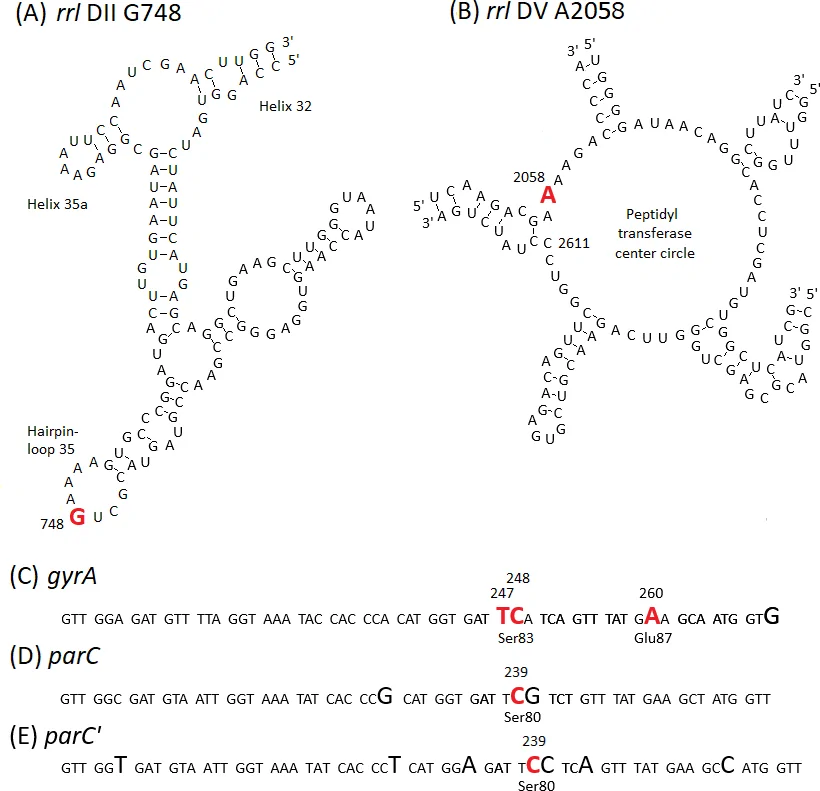
Single nucleotide polymorphisms (SNPs) in target genes associated with decreased antimicrobial susceptibility, as confirmed in *M. bovirhinis* field isolates in Japan. The two-dimensional structure of hairpin-loop 35 in domain II (**A**) and the peptidyl transferase center region in domain V (**B**) of *rrl* of *M. bovirhinis* PG43^T^ (GenBank accession no. LR214972). Each SNP is indicated in large red bold font. The DNA sequences of the quinolone resistance-determining region in *gyrA* (**C**) and *parC* (**D**) of *M. bovirhinis* PG43^T^, and in *parC*′ of *M. bovirhinis* HAZ2616 (**E**). Of the SNPs indicated in large font, those that cause missense mutations are in red bold, and the original amino acid (aa) is shown under the triplet. The nucleotides and aa are numbered based on the *E. coli* sequence (GenBank accession no. NC_002655).

In many *Mycoplasma* species, mutations in genes encoding antimicrobial target factors (e.g., 16S rRNA [*rrs*], 23S rRNA [*rrl*], GyrA, and ParC) are the primary mechanism for decreasing susceptibility (8). In *M. bovis* and *M. californicum*, mutations at *rrl* G748 decrease susceptibility to 16-membered macrolides, and additional mutations at *rrl* A2058 and/or A2059 further decrease susceptibility to 16-membered macrolides (5–7). A total of 48 *M. bovirhinis* field isolates with a mutation at *rrl* G748 and 3 field isolates with mutations at *rrl* G748 and *rrl* A2058 showed significantly higher MIC values for tylosin (MIC: 4‒64 µg/mL [*rrl* G748], 128‒256 µg/mL [*rrl* G748 and *rrl* A2058]) than 49 field isolates without these mutations (MIC: 0.25‒4 µg/mL) (*P* value: <0.0001 [*rrl* G748], *P* value: 0.0034 [*rrl* G748 and *rrl* A2058]) (Fig. 1 and 3). In addition, mutations at *rrl* A2058 and/or A2059 decrease susceptibility to all macrolides and lincosamides in *M. bovis* and *M. californicum* (5–7). Similar to *M. bovis* and *M. californicum*, the three *M. bovirhinis* field isolates carrying the mutation at *rrl* A2058 had significantly higher MIC values for macrolides and lincomycin (MIC _erythromycin_: ≥1,024 µg/mL, MIC _azithromycin_: 512‒>1,024 µg/mL, MIC _lincomycin_: 128‒256 µg/mL) than the 97 *M. bovirhinis* field isolates without this mutation (MIC _erythromycin_: 64‒1,024 µg/mL, MIC _azithromycin_: 2‒64 µg/mL, MIC _lincomycin_: 0.5‒8 µg/mL) (*P* value _erythromycin_: 0.0019, *P* value _azithromycin_: 0.0023, *P* value _lincomycin_: 0.0017) (Fig. 1 and 3). Since the *M. bovirhinis* genome typically contains three *rrs–rrl* operons, there are consequently three target sites for the *rrl* G748 and *rrl* A2058 positions, respectively. The MIC ranges for tylosin in the field isolates carrying *rrl* G748A and *rrl* G748R (excluding the three strains harboring the *rrl* A2058 mutation) were 4–64 µg/mL and 4–32 µg/mL, respectively, and no significant differences were observed. Even among strains harboring *rrl* A2058 mutations, no marked differences were observed in the MICs of azithromycin, tylosin, or lincomycin between field strains carrying *rrl* A2058G and *rrl* A2058W. Fifty-one *M. bovirhinis* field isolates carrying the *rrl* G748 mutation were collected from eight prefectures between 1996 and 2021, and three *M. bovirhinis* field isolates carrying the *rrl* A2058 mutation were collected from Hokkaido Prefecture between 2021 and 2023.

In *M. bovis*, co-missense mutations in GyrA and ParC confer decreased susceptibility to fluoroquinolones. In particular, the co-missense mutations at Ser83 in GyrA and Ser80 in ParC reduce susceptibility to fluoroquinolones more strongly than other combinations of co-missense mutations (6, 7). As mentioned above, many field isolates of *M. bovirhinis* carry ParC′, a ParC homolog, so we screened for missense mutations in both GyrA and ParC and/or ParC′. A missense mutation in GyrA was identified in 98 isolates. In contrast, missense mutations in ParC and/or ParC′ were observed in 34 isolates. Among these, 11 isolates carried a missense mutations in ParC only, 19 in ParC′ only, and 4 in both ParC and ParC′. Overall, all 34 isolates harboring missense mutations in ParC and/or ParC′ also possessed a missense mutation in GyrA. Of the isolates with co-missense mutations in GyrA and ParC and/or ParC′, 33 were mutated at Ser83 in GyrA and Ser80 in ParC and/or ParC′, and the co-missense mutation in the remaining isolate was located at Glu87 in GyrA and Ser80 in ParC′ (Fig. 3). *M. bovirhinis* field isolates with co-missense mutations in GyrA and ParC and/or ParC′ show significantly higher MIC values for enrofloxacin (MIC: 2‒32 µg/mL) than the field isolates without these mutations (MIC: 0.125‒4 µg/mL) (*P* value: <0.0001) (Fig. 1). Incidentally, an isolate with a co-missense mutation at Glu87 in GyrA and at Ser80 in ParC′ showed lower MIC values (2 µg/mL) for enrofloxacin than isolates with a co-missense mutation at Ser83 in GyrA and at Ser80 in ParC′ (8‒32 µg/mL) (Fig. 1). These 33 *M. bovirhinis* field isolates carrying a co-missense mutation at Ser83 in GyrA and at Ser80 in ParC and/or ParC′ were collected from eight prefectures between 2001 and 2021.

A mutation at *rrs* C1192 that decreases susceptibility to spectinomycin has been confirmed in *M. bovis*, but neither low-susceptibility isolates to spectinomycin nor isolates carrying this mutation were detected in *M. bovirhinis* field isolates (6, 7).

The deduced aa sequences of AphA-3 between HAZ141_2 (locus_tag MBVR141_0929, 264 aa) and HAZ2616 (locus_tag MBVR2616_0929, 264 aa) are identical, and identical aa sequences also exist in other bacteria (i.e., *Enterococcus faecium* and *Staphylococcus aureus*; GenBank accession nos. HAQ0809818 and EGQ1519538). In addition to HAZ141_2 and HAZ2616, the presence of *aphA-3* was confirmed in 26 other field isolates, and at least 261 of the 264 amino acids matched the deduced aa sequence of AphA-3 from HAZ141_2, with no deletions, insertions, or extensions observed in these genes (data not shown). We next examined whether *aphA-3* from HAZ141_2 was indeed active and might confer resistance to aminoglycosides (i.e., kanamycin, neomycin, gentamicin, spectinomycin, streptomycin, and nourseothricin) in a heterologous host. To that end, *aphA-3* was cloned into the T-vector pMD20 plasmid derivative (pMD20/*aphA-3*_HAZ141_2) and introduced by transformation into *E. coli* JM109 (see Materials and Methods). No increase in MIC values for gentamicin, spectinomycin, streptomycin, or nourseothricin was observed in the recombinant clone pMD20/*aphA-3*_HAZ141_2 in comparison to the host strain *E. coli* JM109 and *E. coli* transformants carrying an empty pMD20 plasmid vector (Table 1). In contrast, the MIC values for kanamycin and neomycin were at least 64-fold higher in *E. coli* JM109 transformants carrying pMD20/*aphA-3*_HAZ141_2 than in *E. coli* JM109 and the plasmid-containing *E. coli* JM109 recipient, respectively (Table 1). Similar results were obtained for *M. bovirhinis* field isolates: the MIC distributions for kanamycin and neomycin for a total of 28 *aphA-3-*carrying isolates (MIC _kanamycin_: 64‒512 µg/mL, MIC _neomycin_: 256‒>1,024 µg/mL) were significantly higher than those for a total of 72 *aphA-3* non-carrying isolates (MIC _kanamycin_: 1‒16 µg/mL, MIC _neomycin_: 8‒128 µg/mL) (*P* value _kanamycin and neomycin_: <0.0001) (Fig. 1).

**TABLE 1:** Effects on *in vitro* susceptibility to aminoglycosides and oxytetracycline conferred by genes predicted to be involved in decreased antimicrobial susceptibility

	MIC (mg/mL)^*a*^						
Recombinant	Km	Nm	Gm	Spm	Sm	NTC	OTC
JM109	8	16	2	128	16	8	2
JM109 (pMD20)	4	8	2	128	8	8	2
JM109 (pMD20/*aadE*_HAZ2616)	4	8	2	128	1,024	8	2
JM109 (pMD20/aadE*_HAZ141_2)	8	8	2	128	8	8	2
JM109 (pMD20/*sat4*_HAZ141_2)	4	8	2	128	8	≥1,024	2
JM109 (pMD20/*sat4*_HAZ2349)	4	8	2	128	8	1,024	2
JM109 (pMD20/sat4*_HAZ2616)	4	8	2	128	8	8	2
JM109 (pMD20/*aphA-3*_HAZ141_2)	1,024	1,024	2	128	16	16	2
JM109 (pMD20/*rpp*_HAZ2616)	4	8	2	64	8	16	128

^*a*^ Km, kanamycin; Nm, neomycin; Gm, gentamicin; Spm, spectinomycin; Sm, streptomycin; NTC, nourseothricin; OTC, oxytetracycline.

The deduced aa sequence of AadE from HAZ2616 (locus_tag MBVR2616_0546, 305 aa, AadE_HAZ2616) is highly similar to that of *Staphylococcus aureus* (locus_tag SA268_2518, GenBank accession no. AII57085, 302 aa, AadE_Saureus), with three amino acid differences (positions 102, 116, and 131) and a 4-aa C-terminal extension by Trp residues encoded by TGA codons (Fig. S1C). Eleven *M. bovirhinis* field isolates were confirmed to possess amino acid sequences similar to AadE of HAZ2616, and at least 304 of the 305 aa matched among them (data not shown). In AadE of HAZ141_2 (locus_tag MBVR141_0932, 228 aa), the N-terminal 139-aa are replaced with a different 43-aa sequence, three amino acids are different (positions 162, 217, and 258), and the C-terminal 22-aa are extended with Trp residues encoded by TGA codons (Fig. S1C). As with AadE of HAZ141_2, the N-terminal replacement is also confirmed in the homolog of *Streptococcus pyogenes* strain NGAS322 (locus_tag SD89_05780, GenBank accession no. CP010449, 206 aa). AadE of HAZ141_2 and of *S. pyogenes* strain NGAS322, whose functions are presumed to be impaired due to replacement, is marked with an asterisk (AadE*_HAZ141_2, AadE*_Spyogen) (Fig. 1; Fig. S1C; Table 1) (12). Similar aa sequences to AadE* of HAZ141_2 are confirmed in 17 field isolates, and at least 225 of the 228 aa matched among them (data not shown). The gene regions of *aadE* from HAZ141_2 and HAZ2616, excluding the C-terminal extension by Trp residues encoded by TGA codons, were each cloned into the T-vector pMD20 plasmid derivative (pMD20/aadE*_HAZ141_2, pMD20/*aadE*_HAZ2616) and introduced by transformation into *E. coli* JM109. The MIC value for streptomycin was at least 64-fold higher in *E. coli* JM109 transformants carrying pMD20/*aadE*_HAZ2616 than in *E. coli* JM109, the plasmid-containing *E. coli* JM109 recipient, and the transformant carrying pMD20/aadE*_HAZ141_2, respectively. However, no significant changes in susceptibility to other aminoglycosides were observed with the transformants carrying *aadE* or aadE* (Table 1). Similar results were obtained for *M. bovirhinis* field isolates: the MIC distribution for streptomycin for a total of 11 *aadE*-carrying isolates (MIC: 1,024‒>1,024 µg/mL) was significantly higher than that for the 89 *aadE* non-carrying isolates including the 17 aadE*-carrying isolates (MIC: 8‒512 µg/mL) (*P* value: <0.0001) (Fig. 1). Incidentally, although the *rrs* C912T (corresponding to *rrs* T893 in the *M. bovirhinis* PG43ᵀ numbering) has been reported to be associated with decreased susceptibility to streptomycin in *M. capricolum* subsp. *capripneumoniae* (18), the thymine nucleotide was present at this position in all *M. bovirhinis* field strains examined, including PG43ᵀ, regardless of their streptomycin susceptibility.

The deduced aa sequence of Sat4 of HAZ141_2 (MBVR141_0931, 180 aa, Sat4_HAZ141_2) is highly similar to that of *Enterococcus faecium* (GenBank accession no. HAR0326948, 180 aa, Sat4_Efaecium), with only one aa difference between them (position 74), whereas the deduced aa sequence of Sat4 of *Campylobacter jejuni* (GenBank accession no. WP_057098418, 190 aa, Sat4_Cjejuni) contains a 10-aa C-terminal extension (Fig. S1D). Similar aa sequences to Sat4 of HAZ141_2 were confirmed in 16 field isolates, with at least 179 of the 180 amino acids matching among them (data not shown). Sat4 of HAZ2349, one of the field isolates, also has an amino acid sequence similar to Sat4_HAZ141_2, but three amino acids at positions 57 to 59 have been deleted (Fig. S1D). Sat4 of HAZ2616 (MBVR2616_0547, 114 aa) contains a 28-aa deletion from positions 53 to 80, and the same deletion has been identified in Sat4 of *Staphylococcus aureus* (GenBank accession no. HDD6679561, 132 aa). Sat4 of HAZ2616 and that of *S. aureus* are 38-aa and 20-aa shorter at the C-terminus than Sat4 of HAZ141_2, respectively. Sat4 of HAZ2616 and that of *S. aureus*, which are presumed to be functionally impaired due to the deletions, are marked with an asterisk (Sat4*_HAZ2616, Sat4*_Saureus) (Fig. 1; Fig. S1D; Table 1). The gene regions of *sat4* from HAZ141_2, HAZ2369, and HAZ2616 were each cloned into the T-vector pMD20 plasmid derivative (pMD20/*sat4*_HAZ141_2, pMD20/*sat4*_HAZ2369, pMD20/sat4*_HAZ2616) and introduced by transformation into *E. coli* JM109. The MIC value for nourseothricin was at least 128-fold higher in *E. coli* JM109 transformants carrying pMD20/*sat4*_HAZ141_2 and pMD20/*sat4*_HAZ2369 than in *E. coli* JM109 (the plasmid-containing *E. coli* JM109 recipient) and *E. coli* JM109 transformants carrying pMD20/sat4*_HAZ2616, respectively. On the other hand, no effect on MIC values for other aminoglycosides was observed in transformants carrying *sat4* and sat4* (Table 1). Possession of *sat4* is considered a factor contributing to decreased susceptibility to nourseothricin, but no tendency for decreased susceptibility due to possession of *sat4* was observed in *M. bovirhinis* field isolates. The MIC distribution for nourseothricin for the 17 *sat4*-carrying isolates was identical to that for the 83 *sat4*-non-carrying isolates, including the 11 sat4*-carrying isolates (MIC: 4‒32 µg/mL) (*P* value: 0.2464) (Fig. 1).

These field strains harboring aminoglycoside-modifying antimicrobial resistance genes were isolated over a wide geographic area and across extended time periods. Seventeen aadE*-sat4-aphA-3-carrying *M. bovirhinis* field isolates were collected from five prefectures (Hokkaido, Gunma, Nagano, Miyazaki, and Okinawa) between 2001 and 2014, and 11 aadE-sat4*-aphA-3-carrying *M. bovirhinis* field isolates were collected from four prefectures (Hokkaido, Gunma, Miyazaki, and Okinawa) between 2001 and 2023.

In Japan, *M. bovirhinis* field isolates with high MICs for oxytetracycline have been found (Fig. 1). Mutations at *rrs* A965 and *rrs* A967 that decrease susceptibility to tetracyclines have been identified in *M. bovis* (6, 7). This region corresponds to helix 31 of the 16S rRNA component of the 30S subunit of the ribosome, which is involved in binding of tetracyclines (19). However, these mutations were not observed in *M. bovirhinis* field isolates. By contrast, HAZ2616, which has low susceptibility to oxytetracycline, was found to have an Rpp homolog. The deduced aa sequence of Rpp of HAZ2616 (MBVR2616_0528, 639 aa, Rpp_HAZ2616) shows an identity of 77%‒98% (491‒624/639 aa) to TetM/TetW/TetO/TetS family proteins of *Lachnospiraceae* bacterium (GenBank accession no. MBS5128849, 639 aa, Tet_ Lachnospiraceae), *Eubacterium* sp. (GenBank accession no. WP_412962679, 639 aa, Tet_Eubacterium), *Clostridioides difficile* (GenBank accession no. WP_217598232, 639 aa, Tet_Cdifficile), and *Streptococcus suis* (GenBank accession no. HFU3962121, 639 aa, Tet_Ssuis) (Fig. S1E). Incidentally, TetM/TetW/TetO/TetS family proteins from above-mentioned other bacterial species exhibit the highest amino acid sequence identity to the Rpp of strain HAZ2616. The presence of TetM has also been confirmed in *M. hominis* (GenBank accession no. CP011538, 639 aa, Tet_Mhominis) and *U. urealyticum* (GenBank accession no. U08812, 639 aa, Tet_Uurealyticum). These two TetM proteins share a very high amino acid sequence identity with each other (98.1%, 627/639 aa). In contrast, their sequence identities with the Rpp of HAZ2616 were lower, at 69.3% (443/639 aa) for Tet_Mhominis and 68.4% (437/639 aa) for Tet_Uurealyticum (Fig. S1E). Rpp are broadly divided into three groups based on the similarity in their aa sequences (first group: Tet(M), Tet(O), Tet(S) and Tet(W); second group: Otr(A) and TetB(P); third group: Tet(Q) and Tet(T)), and the Rpp carried by *M. bovirhinis* is considered to belong to the first group (20). As with *aphA-3*, *aadE,* and *sat4,* the effect of the *rpp* region from HAZ2616 on oxytetracycline susceptibility was evaluated using an *E. coli* JM109 transformant. The MIC value for oxytetracycline was 64-fold higher in *E. coli* JM109 transformants carrying pMD20/*rpp*_HAZ2616 than in *E. coli* JM109 and the plasmid-containing *E. coli* JM109 recipient (Table 1). Among *M. bovirhinis* field isolates, a total of 17 *rpp*-carrying isolates (MIC: 32‒512 µg/mL) showed a significantly higher MIC distribution for oxytetracycline than the 83 *rpp*‒non-carrying isolates (MIC: 0.5‒16 µg/mL) (*P* value: <0.0001) (Fig. 1). These 17 *rpp*‒carrying *M. bovirhinis* field isolates were collected from four prefectures (Hokkaido, Ōita, Miyazaki, and Okinawa) between 2001 and 2021.

### Rapid detection of genes and mutations associated with decreased susceptibility to antimicrobial agents

**Fig 4:**
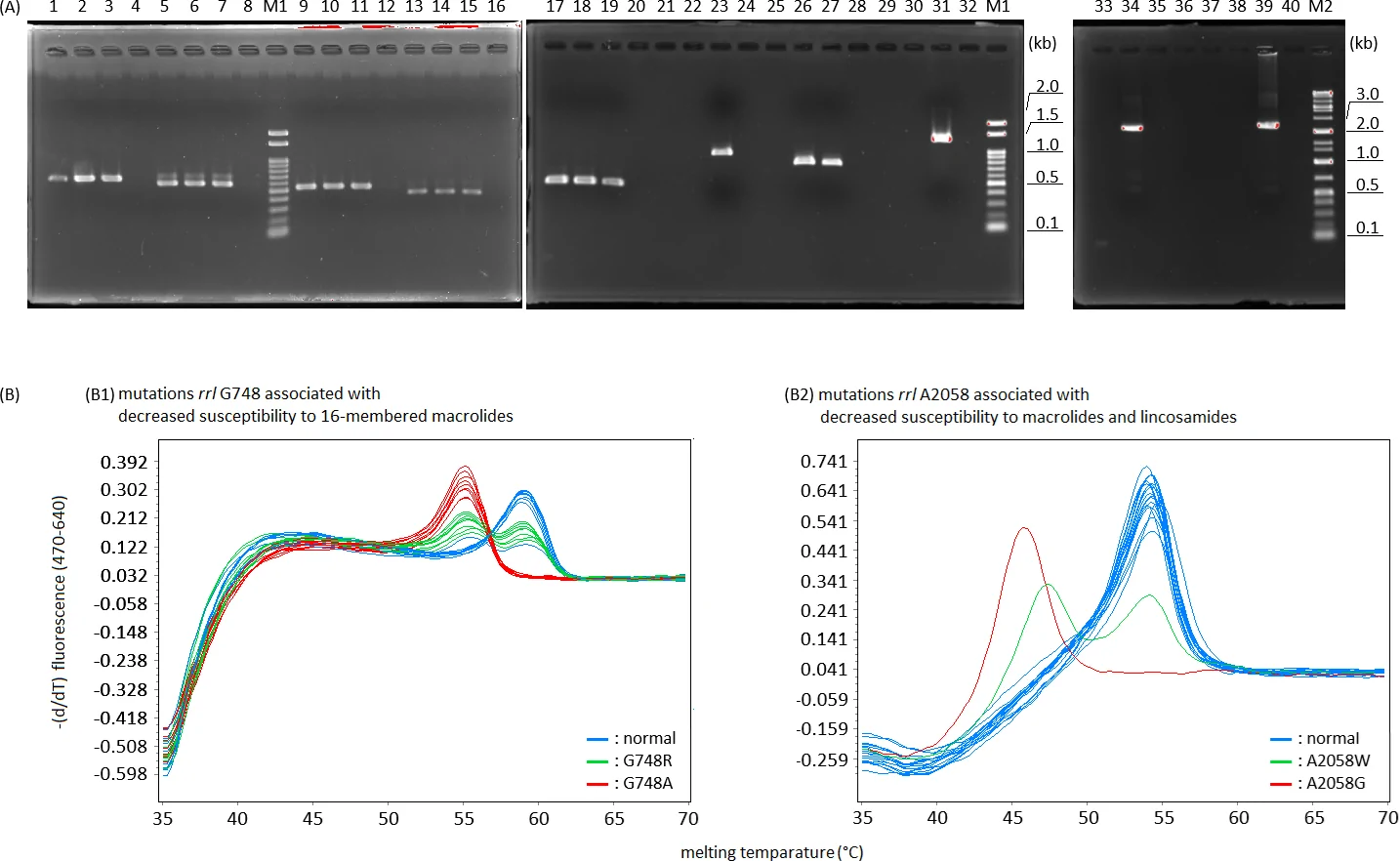
Results of PCR amplification for detecting gene regions associated with decreased susceptibility to antimicrobial agents in *M. bovirhinis*. A total of 2.5 µL of PCR amplicon was applied per lane. Target mutation and gene regions, lanes 1‒4: *rrl* G748, lanes 5‒8: *rrl* A2058, lanes 9‒12: QRDR in *gyrA*, lanes 13‒16: QRDR in *parC*, lanes 17‒20: QRDR in *parC*′, lanes 21‒24: *aadE*, lanes 25‒28: *aphA-3*, lanes 29‒32: *rpp*, lanes 33‒36: aadE*-sat4-aphA-3 cluster, lanes 37‒40: aadE-sat4*-aphA-3 cluster. Tested DNA samples, lanes 1, 5, 9, 13, 17, 21, 25, 29, 33, 37: PG43^T^, lanes 2, 6, 10, 14, 18, 22, 26, 30, 34, 38: HAZ141_2, lanes 3, 7, 11, 15, 19, 23, 27, 31, 35, 39: HAZ2616, lanes 4, 8, 12, 16, 20, 24, 28, 32, 36, 40: negative control (distilled water). Lane M1: Gene Ladder 100 (Nippon Gene), lane M2: Gene Ladder Wide 1 (Nippon Gene) (**A**). The results of melting curve genotyping using hybridization probes for detecting mutations associated with decreased susceptibility to macrolides and lincosamides in *M. bovirhinis* (**B**).

Mutations and genes involved in decreased susceptibility to antimicrobial agents in *M. bovirhinis* field isolates were identified in this investigation (i.e., *rrl* G748 for 16-membered macrolides, *rrl* A2058 for macrolides and lincosamides, co-missense mutations at Ser80 of GyrA and Ser83 of ParC and/or ParC′ for fluoroquinolones, *aphA-3* for kanamycin and neomycin, *aadE* for streptomycin, and *rpp* for tetracyclines). The target DNA regions in field isolates were amplified and sequenced using primers listed in Table S2. Based on the DNA sequences of the target regions in field isolates, PCR primers capable of amplifying these target genes and the mutated regions were designed (Table 2). The species specificity of these primers was examined using DNA solutions from 22 type strains of Mollicutes species reported to be isolated from ruminants (see Materials and Methods). No amplification of the gene regions of *rrl* G748, *rrl* A2058, *gyrA*, and *parC* was observed in any strain other than *M. bovirhinis*. In contrast, primers for the *parC*′ region amplified not only the target region but also a partial region of *gyrA* encoded in *M. verecundum* strain 107 (GenBank accession no. CP137850, locus_tag SAM46_01785). The results of PCR amplification used to detect gene regions associated with decreased antimicrobial susceptibility in *M. bovirhinis* are shown in Fig. 4A. DNA samples used for PCR should generally be prepared from cloned strains rather than directly from lesion material because *aphA-3* and *aadE* are known to be present in species other than *M. bovirhinis*. Furthermore, the presence of a co-infection involving strains harboring missense mutations only in GyrA, only in ParC, and/or only in ParC′ may lead to misdiagnosis.

**TABLE 2:** Primers and probes used in PCR, DNA sequencing, and melting curve genotyping for detecting mutations and genes involved in decreased susceptibility to macrolides, lincosamides, fluoroquinolones, aminoglycosides, and tetracyclines

Mutation or gene	Primer or probes	Sequence (5′ to 3′)	Amplicon size (bp)	Location
*rrl* G748	Mbvr_*rrl* G748_F	GCA GAA ATA GTT GGG AAA C	654	612,291–612,309, 695,341–695,359, 823,572–823,590^*a*^
	Mbvr_*rrl* G748_R	CCG ACT GCA TGA TAA TAC AC		612,925–612,944, 695,975–695,994, 824,206–824,225^*a*^
	Mbvr_*rrl* G748_Dp	GTG GAG GGC CGA ACC GTA GTA-FITC		612,722–612,742, 695,772–695,792, 824,003–824,023^*a*^
	Mbvr_*rrl* G748_Ap	LC Red640-GCT GAA AAG TGC CCG GAT GAC-phosphate		612,744–612,764, 695,794–695,814, 824,025–824,045^*a*^
*rrl* A2058	Mbvr_*rrl* A2058_F	GGT CTT AGG ACT SAA TTG TGT G	595	613,513–613,534, 696,563–696,584, 824,794–824,815^*a*^
	Mbvr_*rrl* A2058_R	CTA GCG CCT TGA TCT CAC TG		614,088–614,107, 697,138–697,157, 825,369–825,388^*a*^
	Mbvr_*rrl* A2058_Dp	GAA AAC GCT GGG TAC CCG CA-FITC		613,988–614,007, 697,038–697,057, 825,269–825,288^*a*^
	Mbvr_*rrl* A2058_Ap	LC Red640-CAA GAC GAA AAG ACC CCA TG-phosphate		614,009–614,028, 697,059–697,078, 825,290–825,309^*a*^
QRDR in *gyrA*	Mbvr_*gyrA_*F	GAG GAT GAT GAA GAA GCT CC	531	255,588–255,607^*a*^
	Mbvr_*gyrA_*R	AAC TAC TGG CTC TTG CTC AG		255,077–255,096^*a*^
QRDR in *parC*	Mbvr_*parC_*F	TCA GCA AAG AGC ATT GCC AG	449	527,802–527,821^*a*^
	Mbvr_*parC*_R	CCA TTC CAA CAG CTA TTC CC		528,231–528,250^*a*^
QRDR in *parC′*	Mbvr_*parC′_*F	GCG AGC CTT GCC TGA TGC AC	447	45,630–45,649^*c*^
	Mbvr_*parC*′_R	GTA GCC ATA CCA ACT GCA ATA C		45,203–45,224^*c*^
*aadE*	Mbvr_*aadE*_F2	TTA GCA GAA CAG GAT GAA CG	936	638,602–638,621^*c*^
	Mbvr_*aadE*_R	CTA TCA CCT CAA ATG GTT CG		639,518–639,537^*c*^
*aphA-3*	Mbvr_*aphA-3*_F	GAG AAT ATC ACC GGA ATT G	745	875,781–875,799^*b*^
	Mbvr_*aphA-3*_R2	CTC CCA ATC AGG CTT GAT CC		875,055–875,074^*b*^
*rpp*	Mbvr_*rpp*_F	TGG CAT TCT TGC ACA CGT TG	1,270	624,079–624,098^*c*^
	Mbvr_*rpp*_R	ATG AGC CTC TTT TCG TGG TC		625,329–625,348^*c*^
aadE*-*sat4*-*aphA-3*	Mbvr_aadE*_F1	CGA TAA ATA AGA ATT TGC GGA GG	2,162	877,073–877,095^*b*^
	Mbvr_*aphA-3*_R1	GAC AGT TGC GGA TGT ACT TC		874,934–874,953^*b*^
aadE-sat4*	Mbvr_*aadE*_F1	GAT GCT GAT TGT ATC GGC	2,398	638,497–638,514^*c*^
-*aphA-3*	Mbvr_ *aphA-3*_R1	GAC AGT TGC GGA TGT ACT TC		640,875–640,894^*c*^

^*a*^ Locations in PG43^T^ genome sequence (GenBank accession no. LR214972).

^*b*^ Locations in HAZ141_2 genome sequence (GenBank accession no. AP018135).

^*c*^ Locations in HAZ2616 genome sequence (GenBank accession no. AP038809).

Mutation detection in *rrl* G748, *rrl* A2058, *gyrA*, and *parC* requires further DNA sequencing analysis using the primers used in PCR. The mutations at *rrl* G748 and *rrl* A2058 can also be detected easily and quickly by melting curve analysis using a hybridization probe. The numbers of *rrs*‒*rrl* operons vary among microbial species, but there are usually three operons in the genome of *M. bovirhinis* (17). In the melting curve analysis using hybridization probes, a single melting peak at a lower-than-normal temperature is observed when mutations occur at the target single nucleotide polymorphisms (SNPs) in all *rrl*s, but the melting curve changes to a bimodal curve if the mutation occurs in only a subset of *rrl*s (Fig. 4B) (7, 21). These changes in the melting curves and peak temperatures were automatically detected and obtained by the real-time PCR instrument’s analysis software. As mentioned earlier, no significant difference in the increase in MIC was observed for any antibacterial agent, regardless of whether the target mutation occurred in all *rrl*s or only in some of them.

## DISCUSSION

The complete genome sequence of *M. bovirhinis* was first reported in 2008 for a Japanese isolate, HAZ141_2, isolated from a nasal swab of a cow (17). In this genome sequence, several aminoglycoside modification genes (aadE*, *sat4*, and *aphA-3*) were collectively encoded in a phage-associated region (12). In lysogenic phages, the prophage is usually integrated into the bacterial genome and is likely to be transmitted vertically during replication (12). Indeed, *M. bovirhinis* strains possessing aminoglycoside-modifying enzyme gene clusters (aadE-sat4-aphA-3* and aadE-sat4-aphA-3*) and *rpp*, as identified in strains HAZ141_2 and HAZ2616, have been widely isolated in Japan since 2001 and are, therefore considered to be already widespread domestically.

A phage-related gene region was also found in HAZ2616, which was isolated in the same year, and the GC content and the presence of TGA codons suggested that this region was clearly derived from another microorganism. This phage-associated region showed a gene sequence very similar to that found in HAZ141_2, including the aminoglycoside-modifying gene cluster (aadE-sat4*-aphA-3). The important differences between the two phage-related gene regions are as follows: *aadE* was intact, *sat4* was partially deleted and non-functional, and *rpp* was additionally encoded.

The *aadE*, *aphA-3*, and *rpp*-carrying isolates were completely consistent with isolates exhibiting low susceptibility to streptomycin, kanamycin, neomycin, and oxytetracycline, and these functions were also reproduced in recombinant *E. coli* strains. AadE and AphA-3 are enzymes that inactivate target antibacterial agents by adenylating the C-6 position of streptidine and phosphorylating the C-3 position of 2-deoxystreptamine, respectively. Therefore, the range of their antibacterial effects is determined by the presence of their target site in aminoglycosides (20, 22).

Rpp is homologous to elongation factors EF-Tu and EF-G, particularly in the N-terminal region containing the GTP-binding domain (23). Rpp inhibits tetracycline antibiotics from binding to ribosomes, thereby conferring antibiotic resistance (19). The higher aa sequence identity of the Rpp of HAZ2616 to TetM/TetW/TetO/TetS family proteins from non-Mollicutes bacteria, compared with TetM from Mollicutes-class microorganisms, suggests that this gene did not originate from Mollicutes. Functional analysis to determine which TetM/TetW/TetO/TetS family Rpp derived from HAZ2616 belongs to remains a future task.

Sat4 inactivates antibiotics such as streptothricin and nourseothricin by acetylating the β-lysine present in these antibiotics (24). The effect of *sat4* from *M. bovirhinis* field isolates was reproduced in recombinant *E. coli*, but no decreased susceptibility to nourseothricin was observed in *sat4*-carrying *M. bovirhinis* field isolates. This phenotypic discrepancy was also reported for the HAZ141_2 strain, which possesses *sat4*, and is speculated to be attributable to the overlap between the 3′-terminal region of aadE* and the 5′-terminal region of *sat4*. Moreover, the existence of a GC-rich GTG start codon for *sat4* might be an additional reason for translational silencing of this gene in mycoplasma cells (12).

Sat4, encoded by several microorganisms, contains a 20-aa homologous region beginning at Leu91 of human spermidine/spermine N1-acetyltransferase (SSAT), and site-directed mutations at Phe94, Val96, Arg101, Gly104, and Gly106 of human SSAT result in a significant loss of function. These data are consistent with the hypothesis that Arg101 and the proximal glycine loop are necessary for the activity of human SSAT (25). This homologous region is also conserved in *sat4* of *M. bovirhinis* and corresponds to Val104, Arg109, Gly112, and Gly114 (see Fig. S1D). Interestingly, this region is also conserved in Sat4*, which has lost the function of Sat4. Incidentally, the deletion site of Sat4*, 28 aa beginning from Glu53, is located 19 aa upstream of these regions.

In *Mycoplasma* species, mutations at *rrl* G748 and *rrl* A2058 have been reported to be key mutation sites involved in decreased susceptibility to macrolides or lincosamides (5–9). The widespread detection of *M. bovirhinis* field strains carrying the *rrl* G748 mutation in Japan is a situation that warrants close attention. In *M. pneumoniae*, the mutation at *rrl* A2058 reportedly has a deleterious effect on growth (26), which may explain its extremely low frequency in *M. bovirhinis* compared to *rrl* G748. A similar tendency has been observed in *M. bovis* (6). Moreover, base pairing between bases 2,057 and 2,611 of *rrl* is a factor that determines susceptibility to 14-membered macrolides and lincosamides; species that do not form base pairs, such as *M. bovirhinis*, are naturally resistant to 14-membered macrolides (e.g., erythromycin) but susceptible to lincosamides (see Fig. 1 and 3). The opposite is true for base-pairing species, such as *M. pneumoniae* and *Ureaplasma* spp (5).

In *M. bovirhinis*, as in *M. bovis*, the coexistence of missense mutations in both GyrA Ser83 and ParC Ser80 appears to be responsible for markedly decreased susceptibility to fluoroquinolones (6, 7). The aa sequences in GyrA Val70-Val90 and ParC Val67-Val87 (corresponding to GyrA Val128-Val148 and ParC Val74-Val94 in the *M. bovirhinis* PG43^T^ numbering, and to GyrA Val137-Val157 and ParC Val78-Val98 in the *M. bovis* PG45^T^ numbering), which are the surrounding regions of GyrA Ser83 and ParC Ser80, are nearly identical between *M. bovirhinis* and *M. bovis* (see Fig. 3; Fig. S1A and B) and are also regions where missense mutations are concentrated in fluoroquinolone-low-susceptibility strains of various *Mycoplasma* species (8). The QRDR in mycoplasmas may be restricted to this region. Many field isolates of *M. bovirhinis* carry a *parC* homolog, *parC*′, and Val67-Val87 in ParC′ were also highly conserved (see Fig. S1A). Most field isolates of *M. bovirhinis* harbor a missense mutation at GyrA Ser83, and missense mutations in not only ParC but also ParC′ contribute to decreased susceptibility to fluoroquinolones. As a result, *M. bovirhinis* may be at higher risk of emerging strains with decreased susceptibility to fluoroquinolones than other *Mycoplasma* species. Indeed, the fact that more than 30% of domestically isolated *M. bovirhinis* field strains possess this combination of missense mutations and are isolated across Japan warrants careful attention.

It was long thought that antimicrobial treatment for *Mycoplasma* infections is not very promising, but in recent years, reports have emerged that support its therapeutic effectiveness (27). The genetic testing-based susceptibility determination method proposed in this paper can provide results in approximately 3 h after preparing the DNA sample of the isolates, thereby enabling the identification of effective antimicrobial agents prior to treatment. We have developed a similar test method for *M. bovis* and *M. californicum* and are introducing it into antimicrobial therapy (5–7). In cases of mammary infection with *M. bovis*, when the causative strain’s susceptibility to fluoroquinolones was confirmed by the genetic testing-based susceptibility determination method, administration of enrofloxacin eliminated *M. bovis* from 80% of infected cows (27). DNA sequencing and melting curve analysis using hybridization probes were used to detect directly point mutations associated with decreased susceptibility. Both methods can clearly detect single-base differences, and the latter allows for detection within 30 min after PCR amplification of the target gene region. Melting curve analysis using hybridization probes carries the risk of detecting point mutations in the vicinity of the target mutation, so if possible, DNA sequencing of the mutated region is recommended for a more accurate assessment (13, 21).

PCR using *parC′* detection primers resulted in nonspecific amplification of *gyrA* in *M. verecundum* 107^T^. In the region corresponding to the Mbvr_parC′_F primer, 17 of the 20 base pairs were identical, with the three mismatched base pairs clustered within 7 base pairs from the 3′ end. Likewise, 20 of the 22 base pairs corresponding to the Mbvr_parC′_R primer were identical in the relevant region. These findings suggest that the high degree of sequence homology at the expected binding sites of the *parC′* detection primers caused the nonspecific amplification.

*Mycoplasma* infection is thought to result in a decline in immune function through increased expression of immune exhaustion-related genes and decreased expression of innate immune response-related genes in mononuclear cells (28). In fact, more than half of cows treated with antimicrobials for *M. bovis*-induced mastitis developed mastitis caused by other bacteria within 2 months after the last antimicrobial treatment; this is presumably due to a persistent decline in immune function even after elimination of *M. bovis* (27). These reports suggest that it is essential to implement hygiene measures to prevent reinfection with the pathogen after antimicrobial treatment of *Mycoplasma* infections. A future challenge is to verify the usefulness of antimicrobial treatment and subsequent hygiene measures in cases of *M. bovirhinis* infection, just as in *M. bovis* infections.

## MATERIALS AND METHODS

### Mycoplasmal isolates, antimicrobial agents, and antimicrobial susceptibility testing

A total of 100 *M. bovirhinis* isolates were collected from bovine samples obtained between 1996 and 2023 in eight prefectures across Japan (Hokkaido, Akita, Gunma, Nagano, Shimane, Ōita, Miyazaki, and Okinawa). The methods used to isolate and identify these isolates have been described previously (29). The susceptibilities of the *M. bovirhinis* isolates to 16 antimicrobials approved for therapeutic use in veterinary medicine in Japan were examined. The following antimicrobials were tested: kanamycin monosulfate, neomycin trisulfate salt hydrate, gentamicin sulfate salt, spectinomycin dihydrochloride pentahydrate, streptomycin sulfate salt, nourseothricin sulfate salt, erythromycin (Sigma-Aldrich Co., St. Louis, MO, USA), azithromycin dihydrate (LKT Laboratories, Inc., St. Paul, MN, USA), tylosin tartrate (MP Biomedicals, LLC, Illkirch, France), lincomycin hydrochloride monohydrate, oxytetracycline hydrochloride, florfenicol, enrofloxacin, tiamulin (Wako Pure Chemical Industries, Ltd., Osaka, Japan), flumequine (Kanto Chemical Co., Inc, Tokyo, Japan), and valnemulin hydrochloride (Tokyo Chemical Industry Co., Ltd., Tokyo, Japan). For quality control of the test and for later evaluation, *M. bovirhinis* PG43^T^ was also added to this study, and the MIC values of the antimicrobial agents against each isolate were determined by the agar microdilution method according to the method recommended by Hannan (14).

### Complete genome sequencing of *M. bovirhinis* HAZ2616

*M. bovirhinis* HAZ2616 was isolated in 2008 from the nasal discharge of Japanese black cattle housed in Miyazaki Prefecture, Japan. After culturing at 37°C for 9 h in 50-mL Hayflick broth (30) with stirring, the bacterial pellet was collected by centrifugation (×12,000 rpm, 4°C for 10 min). The bacterial pellet was suspended in 400 µL of TES buffer (50 mM Tris-HCl [pH 7.5], 5 mM EDTA [pH 8.0], 50 mM NaCl), and total DNA was prepared after phenol‒chloroform extraction and ethanol precipitation, followed by dissolution in 100 µL of TE buffer. The total DNA solution was subjected to sequencing using the PacBio Sequel IIe at the Macrogen Genome Center (Seoul, Republic of Korea). The resulting reads were assembled *de novo* using Microbial Genome Analysis v. SMRTLINK_13.1.0.221,970 to generate one contig, which had a closed-ring structure. DFAST (https://dfast.ddbj.nig.ac.jp/) was used to detect genes in the genome sequence. After performing an initial annotation using the Basic Local Alignment Search Tool (BLAST, 2.14.0), we performed manual curation, followed by verification of potential pseudogenes by PCR and Sanger sequencing. A predicted insertion sequences comparison figure (Fig. 2) between *M. bovirhinis* HAZ141_2 and HAZ2616 was generated using Easyfig (16). Moreover, aa sequence alignment of genes predicted to be involved in decreased antimicrobial susceptibility identified in the genome sequences of *M. bovirhinis* strains HAZ141_2 and HAZ2616 with homologous genes in other bacterial species was performed using CLUSTAL W (Fig. S1) (31).

### Cloning and transformation

To clone aadE*, *sat4*, and *aphA-3* from *M. bovirhinis* strains HAZ141_2 and HAZ2349, the following forward and reverse primers were used for PCR amplification: rAadE*_F and rAadE*_R, rSat4_F and rSat4_R, and rAphA-3_F and rAphA-3_R. To clone *aadE*, sat4*, and *rpp* from *M. bovirhinis* strain HAZ2616, the following forward and reverse primers were used for PCR amplification (Table S1): rAadE_F and rAadE_R, rSat4_F and rSat4_R, and rRpp_F and rRpp_R. PCR amplifications were conducted using the GeneAmp High Fidelity PCR System (Applied Biosystems, Foster City, CA, USA), and the products were purified using a LaboPass Gel Extraction Kit (Cosmo Genetech, Seoul, Republic of Korea) and sequenced on a 3500 Genetic Analyzer (Applied Biosystems) using a BigDye Terminator (version 3.1) ready reaction cycle sequencing kit (Applied Biosystems). The PCR amplification program was as follows: predenaturation at 94°C for 2 min, 10 cycles of (denaturation at 94°C for 15 s, primer annealing at 50°C for 30 s, and extension at 72°C for 60 s per kb of amplicon size), followed by 20 cycles of (denaturation at 94°C for 15 s, primer annealing at 50°C for 30 s, and extension at 72°C for 60 s per kb of amplicon size + 5 s per cycle). The resulting PCR fragment was cloned into the T-Vector pMD20 using the Mighty TA-cloning Kit (TaKaRa Bio, Inc., Kusatsu, Japan). The recombinant clones and the pMD20 plasmid itself were transformed into competent cells of *E. coli* strain JM109 (Nippon Gene Co., Ltd, Toyama, Japan). The transformants were plated on Luria‒Bertani (LB) agar plates containing ampicillin (100 µg/mL). DNA of the recombinant plasmids was isolated using a QuickGene Plasmid Kit S (Fujifilm, Tokyo, Japan) according to the manufacturer’s instructions. After PCR using PrimeSTAR GXL DNA polymerase (TaKaRa Bio, Inc.) with M13 primer M4 and M13 primer RV (Table S1), the orientation of the cloned insertion sequences in pMD20 was confirmed by DNA sequencing with the same primers. The PCR amplification program was as follows: 35 cycles of (denaturation at 98°C for 10 s, primer annealing at 50°C for 15 s, and extension at 68°C for 60 s per kb of amplicon size). The pMD20 derivative products were 3,424 bp (aadE*_HAZ141_2), 3,745 bp (*aadE*_HAZ2616), 3,314 bp (*sat4*_HAZ141_2), 3,305 bp (*sat4*_HAZ2349), 3,093 bp (sat4*_HAZ2616), 3,642 bp (*aphA-3*_HAZ141_2), 4,692 bp (*rpp*_HAZ2616), and 175 bp (no-insert) long and contained the origin of replication as well as the Amp^r^ gene encoding a β-lactamase responsible for ampicillin resistance. The resulting recombinant plasmids were completely sequenced on both strands: pMD20/aadE*_HAZ141_2, pMD20/*aadE*_HAZ2616, pMD20/*sat4*_HAZ141_2, pMD20/*sat4*_HAZ2349, pMD20/sat4*_HAZ2616, pMD20/*aphA-3*_HAZ141_2, and pMD20/*rpp*_HAZ2616). The MICs of transformants were determined according to the Clinical and Laboratory Standards Institute (CLSI) method for performing the agar dilution antimicrobial susceptibility test for bacteria that grow aerobically (32).

### Analysis of genes and mutations associated with decreased susceptibility to antimicrobial agents

*M. bovirhinis* genomic DNA was prepared from 7-mL logarithmic-phase Hayflick broth cultures using an InstaGene matrix (Bio-Rad Laboratories, Hercules, CA, USA) according to the manufacturer’s instructions. PCR amplifications were conducted using PrimeSTAR GXL DNA polymerase. The PCR amplification program was as follows: 35 cycles of (denaturation at 98°C for 10 s, primer annealing at 50°C for 15 s, and extension at 68°C for 60 s per kb of amplicon size). The PCR products were purified using a LaboPass PCR purification kit (Cosmo Genetech) and sequenced on a 3500 Genetic Analyzer using a BigDye Terminator ver. 3.1 ready reaction cycle sequencing kit. Oligonucleotide primers for the PCR amplification and sequencing of target genes (i.e., *rrl*, QRDR in *gyrA*, QRDR in *parC*, QRDR in *parC*′, *aadE*, aadE*, *sat4*, sat4*, *aphA-3*, and *rpp*) listed in Table S2 were commercially synthesized (Hokkaido System Science Co., Ltd., Sapporo, Japan). These primers were designed based on the genome sequences of four *M. bovirhinis* strains: PG43^T^, HAZ141_2, GS01, and HAZ2616 (17). Sequence editing, consensus, and alignment were performed using GENETYX version 13 (Tokyo, Japan). The numbering of nucleotides and aa positions throughout this manuscript is based on the *rrs*, *rrl*, *gyrA*, and *parC* of *Escherichia coli* (33–35) unless otherwise indicated. Using GraphPad PRISM ver. 5.01 (GraphPad Software, Boston, MA, USA), significant differences in MICs were calculated between isolates possessing genes and mutations associated with decreased susceptibility to antimicrobial agents and those without them (Table 1).

### Rapid detection of mutations associated with decreased susceptibility to antimicrobial agents by melting curve analysis using hybridization probes

Oligonucleotide primers and hybridization probes for the detection of target DNA regions (i.e., *rrl* G748, *rrl* A2058, QRDR in *gyrA*, QRDR in *parC*, QRDR in *parC*′, *aadE*, *aphA-3*, *rpp*, aadE*-sat4-aphA-3 cluster, and aadE-sat4*-aphA-3 cluster) were designed based on comparative analysis of the corresponding regions in *M. bovirhinis* field isolates. Oligonucleotide primer sequences for PCR, hybridization probe sequences, and amplicon sizes are shown in Table 2. PCR amplifications were conducted using PrimeSTAR GXL DNA polymerase, with amplification conditions consisting of 35 cycles of (denaturation at 98°C for 10 s, primer annealing at 60°C for 15 s, and extension at 68°C for 60 s per kb of amplicon size). The species specificity of these primers was evaluated using 22 type strains of Mollicutes (i.e., *Acholeplasma axanthum* S-743^T^, *A. granularum* BTS-39^T^, *A. laidlawii* PG8^T^, *A. modicum* PG49^T^, *A. oculi* 19L^T^, *M. agalactiae* PG2^T^, *M. alkalescens* PG51^T^, *M. arginini* G230^T^, *M. bovigenitalium* PG11^T^, *M. bovis* PG45^T^, *M. bovoculi* M165/69^T^, *M. californicum* ST-6^T^, *M. canadense* 275C^T^, *M. capricolum* subsp. *capricolum* California kid^T^, *M. capricolum* subsp. *capripneumoniae* F38^T^, *M. dispar* 462/2^T^, *M. leachii* PG50^T^, *M. mycoides* subsp. *capri* PG3^T^, *M. ovipneumoniae* Y98^T^, *M. putrefaciens* KS1^T^, *M. verecundum* 107^T^, and *M. bovirhinis* PG43^T^).

The melting curve analysis to detect target mutations was carried out in a 20-μL solution of two-fold-diluted PCR product containing 0.2 μM of each hybridization probe. These consisted of a donor probe with its 3′ end labeled with fluorescein isothiocyanate (FITC) and an acceptor probe with its 5′ end labeled with LC Red640. The protocol for the melting curve analysis using a LightCycler480 System II (Roche Diagnostics GmbH, Mannheim, Germany) consisted of a single cycle of 95°C for 60 s (20°C per s), 40°C for 60 s (20°C per s), and 70°C for 0 s (0.1°C per s). The melting curve data were evaluated using the analytical software included with the real-time PCR machine (operated in automatic mode), i.e., LightCycler 480 SW 1.5.1 and Exor4 for XDMS_R (Roche Diagnostics GmbH).

## Supplementary Material

Reviewer comments

## SUPPLEMENTAL MATERIAL

Supplemental figure and tables spectrum.00607-26-s0001.pdf Fig. S1; Tables S1 and S2.

OPEN PEER REVIEW reviewer-comments.pdf An accounting of the reviewer comments and feedback.

ASM does not own the copyrights to Supplemental Material that may be linked to, or accessed through, an article. The authors have granted ASM a non-exclusive, world-wide license to publish the Supplemental Material files. Please contact the corresponding author directly for reuse.
