# Supplementary material for: Identification of genes and mutations involved in antimicrobial low susceptibility in Mycoplasma bovirhinis isolates from Japan and development of a genetic method for antimicrobial susceptibility discrimination
Source: Microbiol Spectr. 2026 Jun 12;14(7):e00607-26. doi: 10.1128/spectrum.00607-26 (PMC13340128; doi:10.1128/spectrum.00607-26)
Supplement: Supplemental figure and tables — Fig. S1; Tables S1 and S2. [file spectrum.00607-26-s0001.pdf]

(A) DNA topoisomerase IV C subunit (ParC)

```
                        10|        20|        30|        40|        50|        60|        70|        80|        90|       100|
ParC_PG43^T     MKNKINQTIE NVVEETLEKT LSERFERYAK YVIQQRALPD VRDGLKPVQR RILYSMFTLG LDPDKPYKKS ARVVGDVIGK YHPHGDSSVY EAMVNLSQWW
ParC_HAZ141_2   MKNKINQTIE NVVEETLEKT LSERFERYAK YVIQQRALPD IRDGLKPVQR RILYSMFTLG LDPDKPYKKS ARVVGDVIGK YHPHGDSSVY EAMVNLSQWW
ParC_GS01       MKNKINQTIE NVVEETLEKT LSERFERYAK YVIQQRALPD VRDGLKPVQR RILYSMFTLG LDPDKPYKKS ARVVGDVIGK YHPHGDSSVY EAMVNLSQWW
ParC'_HAZ2616   MKTKINQTLE KIIDETLEKT LSERFERYAK YVIQQRALPD ARDGLKPVQR RIIYSMFSLG LDFDKPYKKS ARVVGDVIGK YHPHGDSSVY EAMVNMSQWW
                **.*****:* ::::****** ********** **********  ********* **:****:** ** ******* ********** ********** *****:****

                       110|       120|       130|       140|       150|       160|       170|       180|       190|       200|
ParC_PG43^T     KMNLPILDMH GNIGSIDNDP AAQMRYTEVR LSKVAQYMIG DIKKKTTLFV PNFDDSEQEP LVLPSLFPNL LVNGAMGIAV GMATNMLPHN LNEIIDASIY
ParC_HAZ141_2   KMNLPILDMH GNIGSIDNDP AAQMRYTEVR LSKVAQYMIG DIKKKTTLFV PNFDDSEQEP LVLPSLFPNL LVNGAMGIAV GMATNMLPHN LNEVIDASIY
ParC_GS01       KMNLPILDMH GNIGSIDNDP AAQMRYTEVR LSKVAQYMIG DIKKKTTLFV PNFDDSQQEP LVLPSLFPNL LVNGAMGIAV GMATNMLPHN LNEIIDASIY
ParC'_HAZ2616   KNNIPLLDMH GNIGSIDNDP AAQMRYTEVR LSKIAKYMIG DIKKKTTLFV PNFDDSEKEP LVLPTLFPNL LVNGAMGIAV GMATNMLPHN LNEIIDATIH
                * *:*:**** ********** ********** ***:*:**** ********** ******::** ****:***** ********** ********** ***:***:*:

                       210|       220|       230|       240|       250|       260|       270|       280|       290|       300|
ParC_PG43^T     KIKNPQTSLK SLMKYIQGPD FPTGGIIHGT NGIIEGFETG NNSTKNKIKL FSKYNVYTKG QYKYIEITEI PYGVIKSSLV YSIDEIIQNK AIAGLLEIKD
ParC_HAZ141_2   KIKNPQTSLK SLMKYIQGPD FPTGGIIHGT NGIIEGFETG NNSTKNKIKL FSKYNVYTKG QYKYIEITEI PYGVIKSSLV YSIDEIIQNK AIAGLLEIKD
ParC_GS01       KIKNPQTSLK SLMKYIQGPD FPTGGIIHGT NGIIEGFETG NNSTKNKIKL FSKYNVYTKG QYKYIEITEI PYGVIKSSLV YSIDEIIQNK AIAGLLEIKD
ParC'_HAZ2616   KIKNPTASLK SILKYIKGPD FPTGGIIHGT NGIIEAFEYG NNSTKNKIRL FSKYDIYTKG EYKYVEITEI PYGVVKSALV YSIDEIIQNK SIAGLLEVKD
                ***** :*** *::***:*** ********** *****.** * ********:* ****::**** :***:***** ****:**:** ********** :******:**

                       310|       320|       330|       340|       350|       360|       370|       380|       390|       400|
ParC_PG43^T     QSDRSGINIL LVLEKDANDK SIISYLFEKT KLQNTYNYNQ VVIINGRPKL ASLLDLLDNY ILHVKDVKSK VIKFDLEKSK LRLEIIILGLL KVTEITDEVI
ParC_HAZ141_2   QSDRSGINIL LVLEKDANDK SIISYLFEKT KLQNTYNYNQ VVIINGRPKL ASLLDLLDNY ILHVKDVKSK VIKFDLEKSK LRLEIIILGLL KVTEITDEVI
ParC_GS01       QSDRSGINIL LVLEKDANDK SIISYLFEKT KLQNTYNYNQ VVIINGRPKL ASLLDLLDNY ILHVKDVKSK VIKFDLEKSK LRLEIIILGLL KVAEITDEVI
ParC'_HAZ2616   QSDRSGINIL LTLEKDSNDK SIMSYLFTKT KLQNTYNYNQ VVIVNGRPKL ASLLDLLDNY ILHVKEVKTK TITFDLEKSN LRLEIIILGLL RVSEITDAII
                ********** *.****:*** **:**** ** ********** ***:****** ********** *****:**:* .*.******: ********** :*:**** :*

                       410|       420|       430|       440|       450|       460|       470|       480|       490|       500|
ParC_PG43^T     ALIRNCEGSK SGVINALMLS FNFTENQATA IAELRLYKLS KTDKEALLDE KAQLIKNIEH YELLLNDEVK FNEYVINELK SIKKLFGKER KTQIIQEDFN
ParC_HAZ141_2   ALIRNCEGSK SGVINALMLS FNFTENQATA IAELRLYKLS KTDKEALLNE KAQLIKNIEH YELLLNDEVQ FNEYVINELK SIKKLFGKER KTQIIQEDFN
ParC_GS01       ALIRNCEGSK SGVISALMLS FNFTKNQATA IAELRLYKLS KTDKEALLDE KAQLIKNIEH YELLLNDKIQ FNEYIINELK NIKKLFGKER KTQIIQEDFN
ParC'_HAZ2616   SVIRNAQGSK AGVIQALIDN FKFTQNQATA IAELRLYKLS KTDKEVLNQE KEELQKNISL FELLLSNEDK FNGYIINELK EIKKLFGYPR KTQIIEEDYI
                ::***.:*** :***.**: . *:**:***** ********** *****.* :* * :* ***.  :****.:: : ** *:***** .******  * *****:**:

                       510|       520|       530|       540|       550|       560|       570|       580|       590|       600|
ParC_PG43^T     FTYHETDLIK EEVNYVGITK QGFIKKFSDK TVVSNSLSTY PLKDNDVLKY FNKLNSMNTL LIFTNFGNYI QVPVYKINEC KWRETGQKLS DLVHLKVSEE
ParC_HAZ141_2   FTYHETDLIK EEVNYVGITK QGFIKKFSDK TVVSNSLSTY PLKDNDVLKY FNKLNSMNTL LIFTNFGNYI QVPVYKINEC KWRETGQKLS DLVDLKVSEE
ParC_GS01       FTYQETDLIK EEVNYVGITK QGFIKKFSDK TVISNSLSTY PLKDNDVLKY FNKLNSMNTL LIFTNFGSYI QIPVYKINEC KWRETGQKLS DLVDLKVSEE
ParC'_HAZ2616   FTYQETDLIK DVDSYVAITK QGYLKKLSDK TVSSNSLSSY PLKENDFLKY FTKFNSMDTL LIFTTHGNYI QLPVYKINEY KWREAGQKLS ELVDWKLNED
                ***:****** :  .**.*** **::**:*** ** *****:* ***:**.*** *.*:***:** ****..*.** *:*******  ****:***** :**. *:.*:

                       610|       620|       630|       640|       650|       660|       670|       680|       690|       700|
ParC_PG43^T     VVSVIKVDNW NSNTFIVIGT KNGLFKKVKL TEFKVQRLNK VYNAINLTND DKVINVALSN GLYHILILTK NGLCSLYPET DIAIYSPKAK GNKGVYLSLS
ParC_HAZ141_2   VVSVIKVDNW NSNTFIVIGT KNGLFKKVKL TEFKVQRLNK VYNAINLTND DKVINVALSN GLYDILILTK NGLCSLYPET DIAIYSPKAK GNKGVYLSLS
ParC_GS01       VVSVIKVDNW NSNTFIVIGT KNGLFKKVKL NEFKVQRLNK VYNAINLTND DEVINVALSN GLYDILILTK NGLCSLYSET DIAIYSPKAK GNKGVYLSLS
ParC'_HAZ2616   VVSVIKVNNW SEKIFLVIGT KNGLFKKVKL SDFKVQKLNK SYTAINISSG DEVVNVTISN GLHDILILTN NGLCSLYSET DLAIYSPKAK GNKGVYLSLN
                *******:** ..: *:**** ********** .:****:***  *.***:.. *:*:**::** **:.*****: *******.** *:******** *********.

                       710|       720|       730|       740|       750|       760|       770|       780|       790|       800|
ParC_PG43^T     DKVENFAMVQ NNDIVSILTA KGQIKHMLVS KLTYVPKSIK GKKIIEVLES KIIDLCPGYN LKVIGVNGNM QSFLEEINLS SPTKHASITD FNIPSLLEVS
ParC_HAZ141_2   DKVENFAMVQ NNDIVSILTA KGQIKHMLVS KLTYVPKSIK GKKIIEVLES KIIDLCPGYN LKVIGVNGNM QSFLEEINLS SPTKHASITD FNIPSLLEVS
ParC_GS01       DKVKNFAMVQ NNDIVSILTA KGQIKHMLVS KLTYVPKSIK GKKIIEVLES KIIDLCQGYN LKVIGVNGNM QSFLEEINLS SPTKHASITE LNIPSLLEVS
ParC'_HAZ2616   DKVTQFIMVK NQDILNILTS NGQIKHLHVS KLQYVTKLIK GKKIINIDNF KVADLQTGYN FKIIGKNSSN QSFIEELFIP TTIKHGFETD LKIQSLIEIK
                *** :* **: *:**:.***: :*****: ** ** **.* ** *****:: :  *: **  *** :*:** *..  ***:**: :. :. **.  *: ::* **:*:.

                       810|       820|       830|       840|       850|
ParC_PG43^T     FLKLN----- ---------- ---DELDLQK TEQKIKESSL KLEELLKKLN K--
ParC_HAZ141_2   FLKLNDESKS ISTKLFTKES IQNDELDLQK TEQKIKESSL KLEELLKKLN K--
ParC_GS01       FLKLNDESKS ISTKLFTKES IQNDELDLQK TEQKIKESSL KLEELLKKLN K--
ParC'_HAZ2616   FHKLENELDF ETKGLFSKQT LKDEQEDFIR SEAKINESSL RLEELLKKLN NEK
                * **·                   ·· *· · ·* **·**** ·********* ·
```

(B) DNA gyrase A subunit (GyrA)

```
                        10|        20|        30|        40|        50|        60|        70|        80|        90|       100|
GyrA_PG43^T     MQSKFDNEET KKKQLEFEYD HDEDKRVVFK DEIIDQDNNE DDEEAPQNKD DYFAKPQLIS EPVDGLKPVV VDEEMKTSFL EYAMSVIVSR ALPDARDGLK
GyrA_HAZ141_2   MQSKFDNEET KKKQPEFEYD HDEDKRVVFK DEIIDQDNNE DDEEAPQNKD DYFAKPQLIS EPVDGLKPVV VDEEMKTSFL EYAMSVIVSR ALPDARDGLK
GyrA_GS01       MQSKFDDEET KKKQLEFEYD HDEYKRVVFK DEIIDQDNSE DDEEAPQNKD DYFAEPQLIS EPVDGLKPVV VDEEMKTSFL EYAMSVIVSR ALPDARDGLK
GyrA_HAZ2616    MQSKFDNEET KKKQPEFEYD HDEDKRVVFK DEIIDQDNNE DDEEAPQNKD DYFAKPQLIS EPVDGLKPVV VDEEMKTSFL EYAMSVIVSR ALPDARDGLK
                ******:*** **** ***** *** ****** ********.* ********** ****:***** ********** ********** ********** **********

                       110|       120|       130|       140|       150|       160|       170|       180|       190|       200|
GyrA_PG43^T     PVHRRILYDM FELGITPNSQ HRKSARIVGD VLGKYHPHGD SSVYEAMVRM AQDFSMRYPL VDGHGNFGSI DGDQAAAMRY TEARMTKLSN ELLEGIKKDT
GyrA_HAZ141_2   PVHRRILYDM FELGITPNSQ HRKSARIVGD VLGKYHPHGD LSVYEAMVRM AQDFSMRYPL VDGHGNFGSI DGDQAAAMRY TEARMTKLSN ELLEGIKKDT
GyrA_GS01       PVHRRILYDM FELGITPNSQ HRKSARIVGD VLGKYHPHGD LSVYEAMVRM AQDFSMRYPL VDGHGNFGSI DGDQAAAMRY TEARMTKLSN ELLEGIKKDT
GyrA_HAZ2616    PVHRRILYDM FELGITPNSQ HRKSARIVGD VLGKYHPHGD LSVYEAMVRM AQDFSMRYPL VDGHGNFGSI DGDQAAAMRY TEARMTKLSN ELLEGIKKDT
                ********** ********** ********** **********  ********* ********** ********** ********** ********** **********

                       210|       220|       230|       240|       250|       260|       270|       280|       290|       300|
GyrA_PG43^T     VDFVDNYDAT EQEPVVLPAK FPNLLVSGAS GIAVGMATSI PPHNLKEIIN ATIALAKNPE ISTQELMKSV IGPDFPTGGI ILGTKGILDT YETGRGSIIV
GyrA_HAZ141_2   VDFVDNYDAT EQEPVVLPAK FPNLLVSGAS GIAVGMATSI PPHNLKEIIN ATIALAKNPE ISTQELMKSV IGPDFPTGGI ILGTKGILDT YETGRGSIIV
GyrA_GS01       VDFVDNYDAT EQEPVVLPAK FPNLLVSGAS GIAVGMATSI PPHNLKEIIN ATIALAKNPE ISTQELMKSV IGPDFPTGGI ILGTKGILDT YKTGRGSIIV
GyrA_HAZ2616    VDFVDNYDAT EQEPVVLPAK FPNLLVSGAS GIAVGMATSI PPHNLKEIIN ATIALAKNPE ISTQELMKSV IGPDFPTGGI ILGTKGILDT YETGRGSIIV
                ********** ********** ********** ********** ********** ********** ********** ********** ********** *:********

                       310|       320|       330|       340|       350|       360|       370|       380|       390|       400|
GyrA_PG43^T     RSKTQIVENA NGKAKIIVTE IPYAIKTSTI IKKIVELVKE KIIDGIADIR DETNLNGIRI VLDIKKGFSA NIILNQLFQK SDLQTSFNSN LVALVNGEPK
GyrA_HAZ141_2   RSKTQIVENA NGKAKIIVTE IPYAIKTSTI IKKIVELVKE KIIDGIADIR DETNLNGIRI VLDIKKGFST NIILNQLFQK SDLQTSFNSN LVALVNGEPK
GyrA_GS01       RSKTQIVENA NGKAKIIVTE IPYAIKTSTI IKKIVELVKE KIINGIADIR DETNLNGIRI VLDIKKGFSA NIILNQLFQK SDLQTSFNSN LVALVNGEPK
GyrA_HAZ2616    RSKTQIVENA NGKAKIIVTE IPYAIKTSTI IKKIVELVKE KIINGIADIR DETNLNGIRI VLDIKKGFSA NIILNQLFQK SDLQTSFNSN LVALVNGEPK
                ********** ********** ********** ********** ***:****** ********** *********: ********** ********** **********

                       410|       420|       430|       440|       450|       460|       470|       480|       490|       500|
GyrA_PG43^T     TLSLKQALNV YLDHQKEVFT RRLNYDLAKA QEKVHILEGL KVAILNIDEV IAIIKASKTD QIAQEKLMAR FEFTERQTKA ILDMNLRRLT ALNYEKMVNE
GyrA_HAZ141_2   TLSLKQALNV YLDHQKEVFT RRLNYDLAKA QEKVHILEGL KVAILNIDEV IAIIKASKTD QIAQEKLMAR FEFTERQTKA ILDMNLRRLT ALNYEKMVNE
GyrA_GS01       TLSLKQALNV YLDHQKEVFT RRLNYDLAKA QEKVHILEGL KVAILNIDEV IAIIKASKTD QIAQEKLMAR FEFTERQTKA ILDMSLRRLT ALNYEKMVNE
GyrA_HAZ2616    TLSLKQALNV YLDHQKEVFT RRLNYDLAKA QEKVHILEGL KVAILNIDEV IAIIKASKTD QIAQEKLMAR FEFTERQTKA ILDMNLRRLT ALNYEKMVNE
                ********** ********** ********** ********** ********** ********** ********** ********** ****:***** **********

                       510|       520|       530|       540|       550|       560|       570|       580|       590|       600|
GyrA_PG43^T     INELYLEING YQEILSSETK LINLIIEELS IIRDKYGDER RTAIDYYGVG KINDEDLIPK KEIVITASIN GYVKRINLEE YSTQRRGGVG SSSMKTYDDD
GyrA_HAZ141_2   INELYLEING YQEILSSETK LINLIIEELS IIRDKYGDER RTAIDYYGVG KINDEDLIPK KEIVITASIN GYVKRINLEE YSTQRRGGVG SSSMKTYDDD
GyrA_GS01       INELYLEING YQEILSSETK LINLIIEELS IIRDKYADER RTAIDYYGVG KINDEDLIPK KEIVITASIN GYVKRINLEE YSTQRRGGVG SSSMKTYDDD
GyrA_HAZ2616    INELYLEING YQEILSSETK LINLIIEELS IIRDKYGDER RTAIDYYGVG KINDEDLIPK KEIVITASIN GYVKRINLEE YSTQRRGGVG SSSMKTYDDD
                ********** ********** ********** ******.*** ********** ********** ********** ********** ********** **********
```

```
                           610|       620|       630|       640|       650|       660|       670|       680|       690|       700|
GyrA_PG43^T         DISLILKAST HSDLLLFSNF GKAYALRGYQ IPQASKQSKG QPFINVLENL NVLQGEKIIS IINVDSFSDD LFLTTITKKG IFKKTPLSLF ANVRRSGILA
GyrA_HAZ141_2       DISLILKAST HSDLLLFSNF GKAYALRGYQ IPQASKQSKG QPFINVLENL NVLQGEKIIS IINVDSFSDD LFLTTITKKG IFKKTPLSLF ANVRRSGILA
GyrA_GS01           DISLILKAST HSDLLLFSNF GKAYALRGYQ IPQASKQSKG QPFINVLENL NVLQGEKIIS IINVDSFSDD LFLTTITKKG IFKKTPLSLF ANVRRSGILA
GyrA_HAZ2616        DISLILKAST HSDLLLFSNF GKAYALRGYQ IPQASKQSKG QPFINVLENL NVLQGEKIIS IINVDSFSDD LFLTTITKKG IFKKTPLSLF ANVRRSGILA
                    ********** ********** ********** ********** ********** ********** ********** ********** ********** **********

                           710|       720|       730|       740|       750|       760|       770|       780|       790|       800|
GyrA_PG43^T         FKLQDNDQLV RAFIATAGDN ILVANNHKNI ALFNIDSVRA LGRNSLGVKA IKLLEDQYVI NASSNKDGNL IFSLGSKGFG KITHESQYRI TKRGAKGVLG
GyrA_HAZ141_2       FKLQDNDQLV RAFIATAGDN ILVANNHKNI ALFNIDSVRA LGRNSLGVKA IKLLEDQYVI NASSNKDGNL IFSLGSKGFG KITHESQYRI TKRGAKGVLG
GyrA_GS01           FKLQDNDQLV RAFIATAGDN ILVANNHKNI ALFNIDSVRA LGRNSLGVKA IKLLEDQYVI NASSNKDGNL IFSLGSKGFG KITHESQYRI TKRGAKGVLG
GyrA_HAZ2616        FKLQDNDQLV RAFIATAGDN ILVANNNKNI ALFNIDSVRA LGRNSLGVKA IKLLEDQYVI NASSNKDGNL IFSLGSKGFG KITHESQYRI TKRGAKGVLG
                    ********** ********** ******:*** ********** ********** ********** ********** ********** ********** **********

                           810|       820|       830|       840|       850|       860|       870|
GyrA_PG43^T         INTEKSGHLV FASFVNIEDE LLIITASGNT IRIQINQIPQ SSRNTKGVKV INLKNNDQIV AVEVLKNINN
GyrA_HAZ141_2       INTEKSGHLV FASFVNIEDE LLIITSSGNT IRIQINQIPQ SSRNTKGVKV INLKNNDQIV AVEVLKKY--
GyrA_GS01           INIEKSGHLV FASFVNIEDE LLIITSSGNT IRIQINQIPQ SSRNTKGVKV INLKNNDQIV AVEVLKNINN
GyrA_HAZ2616        INTEKSGHLV FASFVNIEDE LLIITASGNT IRIQINQIPQ SSRNTKGVKV INLKNNDQIV AVEVLKNINN
                    ** ******* ********** *****:**** ********** ********** ********** ******:
```

(C) Aminoglycoside 6-adenylyltransferase ANT(6) (AadE)

```
                            10|        20|        30|        40|        50|        60|        70|        80|        90|       100|
AadE*_HAZ141_2      ---------- -----MNQGR IIVITGAPGT GKTTTASAVA KESDLEKSVH MHTDDFYH-- ---------- ---------- ---------- ----------
AadE*_Spyogen       ---------- -----MNQGR IIVITGAPGT GKTTTASAVA KESDLEKSVH MHTDDFYH-- ---------- ---------- ---------- ----------
AadE_HAZ2616        MRSEKEMMDL VLSLAEQDER IRIVTLEGSR ASINIPKDEF QDYDITYFVS DIEPFISNDD WLNQFGNIIM MQKPEDMELF PPEEKGFSYL MLFDDYNKID
AadE_Saureus        MRSEKEMMDL VLSLAEQDER IRIVTLEGSR ANINIPKDEF QDYDITYFVS DIEPFISNDD WLNQFGNIIM MQKPEDMELF PPEEKGFSYL MLFDDYNKID
                                      :: * * ::*    .  .. . ..  :: *:   *       : :

                           110|       120|       130|       140|       150|       160|       170|       180|       190|       200|
AadE*_HAZ141_2      ---------- ---------- ---------- ---------Y HVRKPSAREY DDCCNEFWNV TAYVIKGLCR KEILFAIDHF NQIVRHELLR MISWKVGIET
AadE*_Spyogen       ---------- ---------- ---------- ---------Y HVRKPSAREY DDCCNEFWNV TPYVIKGLCR KEILFAIDHF NQIVRHELLR MISWKVGIET
AadE_HAZ2616        LALLPLEELD NYLKGNKLIK VLIDKDCRIK SDIVPTDIDY HVRKPSAREY DDCCNEFWNV TPYVIKGLCR KEILFAIDHF NQIVRHELLR MISWKVGIET
AadE_Saureus        LTLLPLEELD NYLKGDKLIK VLIDKDCRIK RDIVPTDIDY HVRKPSAREY DDCCNEFWNV TPYVIKGLCR KEILFAIDHF NQIVRHELLR MISWKVGIET
                                                             * ********** ********** *.******** ********** ********** **********

                           210|       220|       230|       240|       250|       260|       270|       280|       290|       300|
AadE*_HAZ141_2      GFKLSVGKNY KFIERYVSED LWEKLLSTYR MDSYENIWEA LFLCHQLFRA VSGEVAEWLH YAYPEYDRNI TKYTRDMYKK YTGKTGCLDS TYAADIEERR
AadE*_Spyogen       GFKLSVGKNY KFIERYISED LWEKLLSTYR MDSYENIWEA LFLCHQLFRA VSGEVAERLH YAYPEYDRNI TKYTRDMYKK YTGKTGCLDS TYAADIEERR
AadE_HAZ2616        GFKLSVGKNY KFIERYISED LWEKLLSTYR MDSYENIWEA LFLCHQLFRA VSGEVAERLH YAYPEYDRNI TKYTRDMYKK YTGKTGCLDS TYAADIEERR
AadE_Saureus        GFKLSVGKNY KFIERYISED LWEKLLSTYR MDSYENIWEA LFLCHQLFRA VSGEVAERLH YAYPEYDRNI TKYTRDMYKK YTGKTGCLDS TYAADIEERR
                    ********** ******:*** ********** ********** ********** ******* ** ********** ********** ********** **********

                           310|       320|
AadE*_HAZ141_2      EQWLQKWKQG TWKISINPAN HLRW
AadE*_Spyogen       EQ-------- ---------- ----
AadE_HAZ2616        EQWLQK---- ---------- ----
AadE_Saureus        EQ-------- ---------- ----
                    **
```

(D) GNAT family N-acetyltransferase (Sat4)

```
                            10|        20|        30|        40|        50|        60|        70|        80|        90|       100|
Sat4_HAZ141_2       MITEMKAGHL KDIDKPSEPF EVIGKIIPRY ENENWTFTEL LYEAPYLKSY QDEEDEEDEE ADCLEYIDNT DKIIYLYYQD DKCVGKVKLR KNWNRYAYIE
Sat4_Cjejuni        MITEMKAGHL KDIDKPSEPF EVIGKIIPRY ENENWTFTEL LYEAPYLKSY QDEEDEEDEE ADCLEYIDNT DKIIYLYYQD DKCVGKVKLR KNWNRYAYIE
Sat4_Efaecium       MITEMKAGHL KDIDKPSEPF EVIGKIIPRY ENENWTFTEL LYEAPYLKSY QDEEDEEDEE ADCLEYIDNT DKIVYLYYQD DKCVGKVKLR KNWNRYAYIE
Sat4_HAZ2349        MITEMKAGHL KDIDKPSEPF EVIGKIIPRY ENENWTFTEL LYEVPFLKSY QDEEDE---E ADCLEYIDNT DKIIYLYYQD DKCVGKVKLR KNWNRYAYIE
Sat4*_Saureus       MITEMKAGHL KDIDKPSEPF EVIGKIIPRY ENENWTFTEL LYEAPYLKSY QD-------- ---------- ---------- DKCVGKVKLR KNWNRYAYIE
Sat4*_HAZ2616       MITEIKAGHL KDIDKPSEPF EVIGKIIPRY ENENWTFTEL LYEAPYLKSY QD-------- ---------- ---------- DKCVGKVKLR KNWNRYAYIE
                    ****:***** ********** ********** ********** ***.*:**** **                               ********** **********

                           110|       120|       130|       140|       150|       160|       170|       180|       190|
Sat4_HAZ141_2       DIAVCKDFRG QGIGSALINI SIEWAKHKNL HGLMLETQDN NLIACKFYHN CGFKIGSVDT MLYANFENNF EKAVFWYLRF ----------
Sat4_Cjejuni        DIAVCKDFRG QGIGSALINI SIEWAKHKNL HGLMLETQDN NLIACKFYHN CGFKIGSVDT MLYANFENNF EKAVFWYLRF FVIISFLGYL
Sat4_Efaecium       DIAVCKDFRG QGIGSALINI SIEWAKHKNL HGLMLETQDN NLIACKFYHN CGFKIGSVDT MLYANFENNF EKAVFWYLRF ----------
Sat4_HAZ2349        DIAVCKDFRG QGIGSALINI SIEWAKHKNL HGLMLETQEN NLIACKFYHN CGFKIGSVDT MLYANFENNF EKAVFWYLRF ----------
Sat4*_Saureus       DIAVCKDFRG QGIGSALINI SIEWAKHKNL HGLMLETQDN NLIACKFYHN CGFKIGSVDT ---------- ---------- ----------
Sat4*_HAZ2616       DIAVCKDFRG QGIGSALINI SIEWAKHKNL HGLMLEPRTI TL-------- ---------- ---------- ---------- ----------
                    ********** ********** ********** ******.:   .*
```

(E) Tetracycline ribosomal protection protein (Rpp)

```
                            10|        20|        30|        40|        50|        60|        70|        80|        90|       100|
Rpp_HAZ2616         MKIINIGILA HVDAGKTTLT ESLLYTSGAI MEPGNVDKGT TRTDSMALER QRGITIQAAV TSFHWKEYKV NLVDTPGHMD FMTEVYRSLS VLDGAILVIS
Tet_Lachnospiraceae MKIINIGILA HVDAGKTTLT ESLLYTSGAI MEPGSVDKGT TRTDSMALER QRGITIQAAV TSFHWKEYKV NLVDTPGHMD FMTEVYRSLS VLDGAILVIS
Tet_Eubacterium     MKIINIGILA HVDAGKTTLT ESLLYTSGAI IEPGSVDKGT TRTDSMALER QRGITIQAAV TSFHWKEYKV NLVDTPGHMD FLTEVYRSLS VLDGAILVVS
Tet_Cdifficile      MKIINIGILA HVDAGKTTLT ESLLYTSGAI TEQGSVDKGT TRTDTMILER QRGITIQTAV TSFCWNGYKI NIVDTPGHMD FLAEVYRSLS VLDGAILVVS
Tet_Ssuis           MKIINIGILA HVDAGKTTLT ESLLYTSGAV PELGSVDKGT TRTDTMLLER QRGITIQTAV TSFGWKNYKI NIVDTPGHMD FLAEVYRSLA VLDGAILVVS
Tet_Mhominis        MKIINIGVLA HVDAGKTTLT ESLLYNSGAI TELGSVDKGT TRTDNTLLER QRGITIQTGI TSFQWENTKV NIIDTPGHMD FLAEVYRSLS VLDGAILLIS
Tet_Uurealyticum    MKIINIGVLA HVDAGKTTLT ESLLYNSGAI TELGSVDKGT TRTDNTLLER QRGITIQTGI TSFQWENTKV NIIDTPGHMD FLAEVYRSLS VLDGAILLIS
                    *******:** ********** *****.***:  * *.***** ****.  *** *******:.: *** *:  *: *::******* *::******: *******::*

                           110|       120|       130|       140|       150|       160|       170|       180|       190|       200|
Rpp_HAZ2616         AKDGVQAQTS VLFHALRKMK IPTIIFVNKI DQNGIDLRRI YQNIREKLTS DMIVMQEVVL SSKITMTDIS DLDKWDTVIA GNDNLLEKYL SGAPLTLREL
Tet_Lachnospiraceae AKDGVQAQTR VLFHALRKMK IPTIIFVNKI DQEGIDLQSV YQNIREKLSD DVMVMQDVSL TPEVSLTDIE DIEKWDSIIA GNDNLLEKYL SGAPLTLREL
Tet_Eubacterium     AKDGVQAQTR VLFHALRKMK IPTIIFINKI DQEGFVPAQT YQNIREKLTE DMMVMQEVHL FSELTLSDVA DFEKWDAVIA GNDDLLEKYL SGAPLTLREL
Tet_Cdifficile      AKDGVQAQTR ILFHALQKMN IPTIIFINKI DQEGFVPAQT YQNIREKLTE DMMVMQEVHL FSELTLSDVA DFEKWDAVIA GSDELLERYI AEGSLDMQEL
Tet_Ssuis           AKDGVQAQTR VLFHALQKMK IPTIIFINKI DQEGFVPAQT YQNIREKLTE DMMVMQEVHL FSELTLSDVV DFEKWDAVIA GNDELLEKYI AGEPLKIQDL
Tet_Mhominis        AKDGVQAQTR ILFHALRKMG IPTIFFINKI DQNGIDLSTV YQDIKEKLSA EIVIKQKVEL YPNMCVTNFT ESEQWDTVIE GNDDLLEKYM SGKSLEALEL
Tet_Uurealyticum    AKDGVQAQTR ILFHALRKMG IPTIFFINKI DQNGIDLSTV YQDIKEKLSA EIVIKQKVEL YPNMCVTNFT ESEQWDTVIE GNDDLLEKYM SGKSLEALEL
                    *********  :*****:**  ****:*:*** **:*:      **:*:***:  :::: *.* *  .::-:::.- :-::**::*  *.*:***:*: :  .*   :*

                           210|       220|       230|       240|       250|       260|       270|       280|       290|       300|
Rpp_HAZ2616         QEEIKRRVQQ GTFFPVYHGS AKENIGTKKL LDVITDIFSS KTDNCQSELC GYVFKIEYTE QKKRRCYLRL YSGTLCLRET ILLSKKEKIK ITEMSIPFDG
Tet_Lachnospiraceae QEEIKRRVQQ GTFFPVYHGS AKENIGTKKL LDVITDIFSS KTDNCQSELC GYVFKIEYTE QKKRRCYLRL YSGTLCLRET ILLSKKEKIK ITEMSIPFDG
Tet_Eubacterium     QEEIKRRVQQ GTFFPVYHGS AKENIGTKEL LDVITDIFSS KTDNCQSELC GYVFKIEYTE QKKRRCYVRL YSGTLCLRET IFLQKKEKIK ITEMSIPFKG
Tet_Cdifficile      QREKCRRTRC CSLFPVYHGS AKDNLGMEKL IEVITETFVP ETENSQSELC GYVFKIEYTD RKKRLSYLRL YHGTLHLRDT IQLSKKKRIK ITEMFIPSNG
Tet_Ssuis           QREKCRRMQN GSLFPIYHGS AKNNIGTEKL IEVIAETFTS GADNDQSELC GSVFKIEYTA QKKRLVYLRL YSGTLHLRDT ILLPQNQKLK ITEMRIPSNG
Tet_Mhominis        EQEESIRFQN CSLFPVYHGS AKNNIGIDNL IEVITNKFYS STHRGQSELC GKVFKIEYSE KRQRLAYIRL YSGVLHLRDL VRISEKEKIK ITEMYTSING
Tet_Uurealyticum    EQEESIRFHN CSLFPVYHGS AKNNIGIDNL IEVITNKFYS STHRGPSELC GNVFKIEYTK KRQRLAYIRL YSGVLHLRDS VRVSEKEKIK VTEMYTSING
                    :.*   * :   ::**:**** **:*:* .:* ::**:: * .  :..  **** * ******:  :::*  *:** * *.* **:  : : :::::* :***  . .*
```

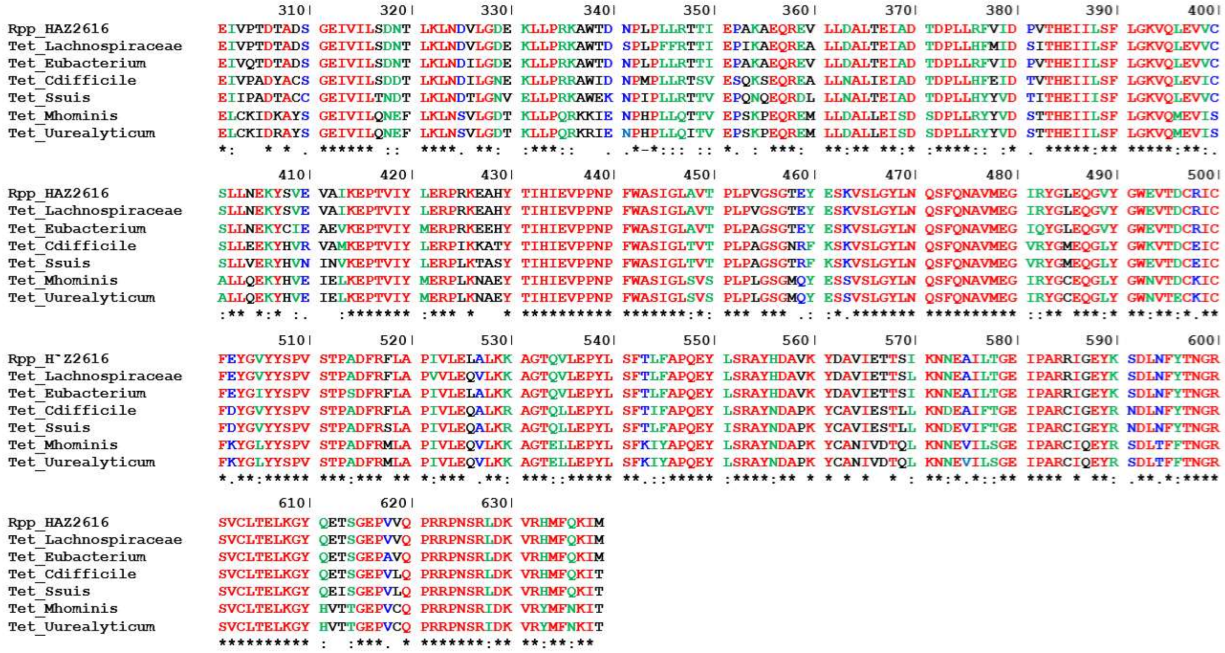

FIG S1 The amino acid (aa) sequence alignment of DNA topoisomerase IV C subunit (ParC) (A) and DNA gyrase A subunit (GyrA) (B) identified in the genome sequences of *M. bovirhinis* strains PG43^{T}, HAZ141_2, GS01, and HAZ2616 (GenBank accession nos. LR214972, AP018135, CP024049, AP038809). The alignment length is 853 aa (ParC) and 870 aa (GyrA) residues; the aa sequences were aligned using CLUSTAL W (31). Identical aa are shown in red and marked with an asterisk (*); strongly similar aa are shown in green and marked with a colon (:); weakly similar aa are shown in blue and marked with a dot (.); different aa are indicated in black. Locations at the 80th serine (=S) of ParC and ParC', the 83rd serine (=S) and the 87th glutamic acid (=E) of GyrA in *E. coli* numbering correspond to the 87th S of ParC and ParC' and the 141st S and the 145th E of GyrA in *M. bovirhinis* numbering. The aa sequence alignment of genes predicted to be involved in decreased antimicrobial susceptibility as identified in the genome sequences of *M. bovirhinis* strains HAZ141_2 and HAZ2616 with homologous genes in other bacterial species. In addition, the names of genes whose functions are presumed to be impaired due to deletion or other reasons are marked with an asterisk (*). The alignment lengths of Aminoglycoside 6-adenylyltransferase (AadE) (C), GNAT family N-acetyltransferase (Sat4) (D), and Tetracycline ribosomal protection proteins (Rpp)

(E) are 324 aa, 190 aa, and 639 aa residues, respectively. The comparison includes AadE*_HAZ141_2 (MBVR141_0932, GenBank accession no. AP018135, 228 aa), *Mycoplasma bovirhinis* strain HAZ141_2; AadE*_Spyogen (SD89_05780, GenBank accession no. CP010449, 206 aa), *Streptococcus pyogenes* strain NGAS322; AadE_HAZ2616 (MBVR2616_0546, GenBank accession no. AP038809, 306 aa), *M. bovirhinis* strain HAZ2616; AadE_Saureus (SA268_2518, GenBank accession no. AII57085, 302 aa), *Staphylococcus aureus* strain SA268. A 22-aa C-terminal extension of AadE*_HAZ141_2 and a 4-aa C-terminal extension of AadE_HAZ2616 are highlighted in yellow, and five tryptophan (=W) residues encoded by TGA codons are underlined (C). The comparison includes Sat4_HAZ141_2 (MBVR141_0931, 180 aa), *M. bovirhinis* strain HAZ141_2; Sat4_Cjejuni (GenBank accession no. WP_057098418, 190 aa), *Campylobacter jejuni*; Sat4_Efaecium (GenBank accession no. HAR0326948, 180 aa), *Enterococcus faecium*; Sat4_HAZ2349 (177 aa), *M. bovirhinis* strain HAZ2349; Sat4*_Saureus (GenBank accession no. HDD6679561, 132 aa), *Staphylococcus aureus*; Sat4*_HAZ2616 (MBVR2616_0547, 114 aa), *M. bovirhinis* strain HAZ2616 (D). The comparison includes Rpp_HAZ2616 (MBVR2616_0528, 639 aa), *M. bovirhinis* strain HAZ2616; Tet_Lachnospiraceae (GenBank accession no. MBS5128849, 639 aa), Lachnospiraceae bacterium; Tet_Eubacterium (GenBank accession no. WP_412962679, 639 aa), *Eubacterium* sp.; Tet_Cdifficile (GenBank accession no. WP_217598232, 639 aa), *Clostridioides difficile*; Tet_Ssuis (GenBank accession no. HFU3962121, 639 aa), *Streptococcus suis*; Tet_ Mhominis (GenBank accession no. CP011538, 639 aa) *Mycoplasma hominis*; Tet_Uurealyticum (GenBank accession no. U08812, 639 aa) *Ureaplasma urealyticum* (E). Incidentally, the aa sequences of Aminoglycoside 3'-phosphotransferase APH(3')-IIIa (AphA-3) in HAZ141_2 (MBVR141_0929, 264 aa) and HAZ2616 (MBVR2616_0548, 264 aa) are identical.

Table S1 Primers for generating transformants to evaluate genes predicted to be involved in decreased susceptibility to antimicrobial agents.

Primer	Sequence (5’ to 3’)	Amplicon size (bp)	Location
rAadE_F	GAT GCT GAT TGT ATC GGC	1,007	638,497-638,514[b]
rAadE_R	ATC TTT CAG GTG CCC TGC		639,486-639,503[b]
rAadE*_F	CGA TAA ATA AGA ATT TGC GGA GG	686	877,073-877,095[a]
rAadE_R	Same as rAadE_R		876,410-876,427[a]
rSat4_F	GCG GGA ACA GTG ATT ACA G	576	876,436-876,454[a]
rSat4_R	CTC CAA TTC ACT GTT CCT TGC		875,879-875,899[a]
rSat4*_F	Same as rSat4_F	355	639,459-639,477[b]
rSat4*_R	GCT ATA AGG TTA TTG TCC TGG G		639,792-639,813[b]
rAphA-3_F	CTG TAG AAA AGA GGA AGG	904	875,820-875,837[a]
rAphA-3_R	GAC AGT TGC GGA TGT ACT TC		874,934-874,953[a]
rRpp_F	GTG AAT GGA GGA TGA CAC ATG	1,954	624,044-624,064[b]
rRpp_R	TGC ATT GCG CAA GAC GTT AC		625,978-625,997[b]
M13 primer M4	GTT TTC CCA GTC ACG AC	176 + cloned insert	51-67[c]
M13 primer RV	CAG GAA ACA GCT ATG AC		2,630-2,646[c]

[a]Locations in HAZ141_2 genome sequence (GenBank accession no. AP018135), [b]locations in HAZ2616 genome sequence (GenBank accession no. AP038809), [c]locations in pMD20 whole sequence (https://www.takarabio.com/products/cloning/linkers-primers-and-cloning-vectors/cloning-vectors/t-vectors-pmd20-and-pmd19).

Table S2 Primers for genetic analysis of genes predicted to be involved in decreased susceptibility to antimicrobial agents.

Gene	Primer[a]	Sequence (5' to 3')	Amplicon size (bp)	Location
rrl	Mbvr_*rrl*_Fp	CGT GAT TAC CTG CGA TAA GC	2,679	612,014-612,033, 695,064-695,083, 823,295-823,314[b]
	Mbvr_*rrl*_Rp	TCC GTA CTT AGC TAC CCA GC		614,673-614,692, 697,723-697,742, 825,954-825,973[b]
	Mbvr_*rrl* G748_Fs	Same as Mbvr_*rrl* G748_F in Table 2		612,291-612,309, 695,341-695,359, 823,572-823,590[b]
	Mbvr_*rrl* G748_Rs	Same as Mbvr_*rrl* G748_R in Table 2		612,925-612944, 695,975-695,994, 824,206-824,225[b]
	Mbvr_*rrl* A2058_Fs	Same as Mbvr_*rrl* A2058_F in Table 2		613,513-613534, 696,563-696,584, 824,794-824,815[b]
	Mbvr_*rrl* A2058_Rs	Same as Mbvr_*rrl* A2058_R in Table 2		614,088-614107, 697,138-697,157, 825,369-825,388[b]
QRDR in *gyrA*	Mbvr_*gyrA*_F1p	YGACGAGKACAAAAGAGTAG	531-2,337	255,643-255,662[b]
	Mbvr_*gyrA*_F2p	Same as Mbvr_*gyrA*_F in Table 2		255,588-255,607[b]
	Mbvr_*gyrA*_R1p	CCT AAA ACR CCT TTA GCA CC		253,326-253,345[b]
	Mbvr_*gyrA*_R2p	Same as Mbvr_*gyrA*_R in Table 2		255,077-255,096[b]
QRDR in *parC*	Mbvr_*parC*_F1p	CTG GGT GAT GCA GGA GAT CG	449-2,902	527,566-527,585[b]
	Mbvr_*parC*_F2p	GAA AAG ACT CTT TCA GAA CG		527,755-527,774[b]
	Mbvr_*parC*_F3p	Same as Mbvr_*parC*_F in Table 2		527,802-527,821[b]
	Mbvr_*parC*_R1p	CTT CCG GCT ACT GCT ACT TC		530,391-530,410[b]
	Mbvr_*parC*_R2p	CGT CTG ACT AAT TCT TGT TCT GTG		530,228-530,251[b]
	Mbvr_*parC*_R3p	Same as Mbvr_*parC*_R in Table 2		528,231-528,250[b]
QRDR in *parC'*	Mbvr_*parC'*_F1p	GCG GGT AGC TTT TAT TGG TCC	447-2,870	46,000-46,020[d]
	Mbvr_*parC'*_F2p	Same as Mbvr_*parC'*_F in Table 2		45,630-45,649[d]
	Mbvr_*parC'*_R1p	CGT CTG ACA AGT TCT TGT TC		43,151-43,170[d]
	Mbvr_*parC'*_R2p	Same as Mbvr_*parC'*_R in Table 2		45,203-45,224[d]
aadE	AadE_Fp	Same as rAadE_F in Table S1	1,007	638,497-638,514[d]
	AadE_Rp	Same as rAadE_R in Table S1		639,486-639,503[d]
	AadE_Fs	CAG ATT GTT CGC CAT GAG C		639,106-639,124[d]
	AadE_Rs	GCT CAT GGC GAA CAA TCT G		639,106-639,124[d]
*aadE**	AadE*_Fp	Same as rAadE*_F in Table S1	686	877,073-877,095[c]
	AadE*_Rp	Same as rAadE_R in Table S1		876,410-876,427[c]
	AadE*_Fs	Same as rAadE_Fs		876,789-876,807[c]
	AadE*_Rs	Same as rAadE_Rs		876,789-876,807[c]
sat4	Sat4_Fp	Same as rSat4_F in Table S1	576	876,436-876,454[c]
	Sat4_Rp	Same as rSat4_R in Table S1		875,879-875,899[c]

*sat4**	Sat4*_Fp	Same as rSat4_F in Table S1	355	639,459-639,477[d]
	Sat4*_Rp	Same as rSat4*_R in Table S1		639,792-639,813[d]
aphA-3	AphA-3_Fp	Same as rAphA-3_F in Table S1	904	875,820-875,837[c]
	AphA-3_Rp	Same as rAphA-3_R in Table S1		874,934-874,953[c]
	AphA-3_Fs	GAT TAT CGA GCT GTA TGC		875,464-875,481[c]
	AphA-3_Rs	AAG AGC CTG ATG CAC TCC		875,446-875,463[c]
rpp	Rpp_Fp	Same as rRpp_F in Table S1	1,954	624,044-624,064[d]
	Rpp_Rp	Same as rRpp_R in Table S1		625,978-625,997[d]
	Rpp_F1s	TGA TTG ATC CTG TCA CTC		625,194-625,211[d]
	Rpp_R1s	CAC AGT TCC GAT TGA CAG		624,793-624,810[d]
	Rpp_R2s	Same as Mbvr_*Rpp*_R in Table 2		625,329-625,348[d]

[a]Primer names ending in "p" are for PCR and DNA sequencing. Primer names ending in "s" are for DNA sequencing. [b]Locations in PG43^T genome sequence (GenBank accession no. LR214972), [c]locations in HAZ141_2 genome sequence (GenBank accession no. AP018135), [d]locations in HAZ2616 genome sequence (GenBank accession no. AP038809).
