# Supplementary material for: Identification of genes and mutations involved in antimicrobial low susceptibility in Mycoplasma bovirhinis isolates from Japan and development of a genetic method for antimicrobial susceptibility discrimination
Source: Microbiol Spectr. 2026 Jun 12;14(7):e00607-26. doi: 10.1128/spectrum.00607-26 (PMC13340128; doi:10.1128/spectrum.00607-26)
Supplement: Reviewer comments [file reviewer-comments.pdf]

AMERICAN SOCIETY FOR MICROBIOLOGY | Microbiology Spectrum

Identification of genes and mutations involved in antimicrobial low susceptibility in *Mycoplasma bovirhinis* isolates from Japan and development of a genetic method for antimicrobial susceptibility discrimination

Eiji Hata

Corresponding Author(s): Eiji Hata, Nogyo Shokuhin Sangyo Gijutsu Sogo Kenkyu Kiko Dobutsu Eisei Kenkyu Bumon

Review Timeline:

Submission Date:	February 18, 2026
Editorial Decision:	March 14, 2026
Revision Received:	May 11, 2026
Accepted:	May 12, 2026

Editor: Artem Rogovsky

Reviewer(s): The reviewers have opted to remain anonymous.

Transaction Report:

(Note: With the exception of the correction of typographical or spelling errors that could be a source of ambiguity, letters and reports are not edited. The original formatting of letters and referee reports may not be reflected in this compilation.)

DOI: https://doi.org/10.1128/spectrum.00607-26

Re: Spectrum00607-26 (Identification of genes and mutations involved in antimicrobial low susceptibility in Mycoplasma bovirhinis isolates from Japan and development of a genetic method for antimicrobial susceptibility discrimination)

Dear Dr. Eiji Hata:

Thank you for the privilege of reviewing your work. Below you will find my comments, instructions from the Spectrum editorial office, and the reviewer comments.

Please return the manuscript within 60 days; if you cannot complete the modification within this time period, please contact me. If you do not wish to modify the manuscript and prefer to submit it to another journal, notify me immediately so that the manuscript may be formally withdrawn from consideration by Spectrum.

Revision Guidelines
To submit your modified manuscript, log into the submission site at https://spectrum.msubmit.net/cgi-bin/main.plex. Go to Author Tasks and click the appropriate manuscript title to begin. The information you entered when you first submitted the paper will be displayed; update this as necessary. Note the following requirements:

• Upload point-by-point responses to the issues raised by the reviewers in a file named "Response to Reviewers," NOT in your cover letter.
• Upload a compare copy of the manuscript (without figures) as a "Marked-Up Manuscript" file.
• Upload a clean .DOC/.DOCX version of the revised manuscript and remove the previous version.
• Each figure must be uploaded as a separate, editable, high-resolution file (TIFF or EPS preferred), and any multipanel figures must be assembled into one file.
• Any supplemental material intended for posting by ASM should be uploaded with their legends separate from the main manuscript. You can combine all supplemental material into one file (preferred) or split it into a maximum of 10 files with all associated legends included.

For complete guidelines on revision requirements, see our Submission and Review Process webpage. Submission of a paper that does not conform to guidelines may delay acceptance of your manuscript.

Data availability: ASM policy requires that data be available to the public upon online posting of the article, so please verify all links to sequence records, if present, and make sure that each number retrieves the full record of the data. If a new accession number is not linked or a link is broken, provide Spectrum production staff with the correct URL for the record. If the accession numbers for new data are not publicly accessible before the expected online posting of the article, publication may be delayed; please contact production staff (Spectrum@asmusa.org) immediately with the expected release date.

Publication Fees: For information on publication fees and which article types are subject to charges, visit our website. If your manuscript is accepted for publication and any fees apply, you will be contacted separately about payment during the production process; please follow the instructions in that e-mail. Arrangements for payment must be made before your article is published.

ASM Membership: Corresponding authors may join or renew ASM membership to obtain discounts on publication fees. Need to upgrade your membership level? Please contact Customer Service at Service@asmusa.org.

The ASM Journals program strives for constant improvement in our submission and publication process. Please tell us how we can improve your experience by taking this quick Author Survey.

Thank you for submitting your paper to Spectrum.

Sincerely,
Artem Rogovsky
Editor
Microbiology Spectrum

Reviewer #1 (Comments for the Author):

The manuscript by Hata is an extensive analysis of the antimicrobial susceptibility patterns and underlying resistance mechanisms in a large collection (100 isolates) of Mycoplasma bovirhinis strains from Japan. Molecular methods for identifying susceptibility-related point mutations in housekeeping genes is also developed. The text is very clear and I have only a few minor comments, mainly editorial in nature, listed below.

Specific points (mainly minor language related)
1. L11 Perhaps add "antimicrobial" to the running head
2. L20 and L44 At least on first mention in each section, please add the new name Mycoplasmopsis bovirhinis, in parentheses, after the Mycoplasma name
3. L51, L55, L63, L65, L415, L439, L451 and elsewhere if necessary. Please italicize "mycoplasma"
4. L62 Perhaps use "should lead to the "
5. L66 takes 2 or more days.
6. L78 methods is important
7. L88 Please define MIC on first mention
8. L155 to certain antimicrobial agents
9. L309helix 31 of the 16S rRNA component of the 30S subunit
10. L313 What does the size range here mean? The extent of homologous regions or different sizes of the various Tet family proteins?
11. L313 Please specifically state that these are the the highest identity matches. Plesae also see pt 17 below.
12. L313 I think it would be useful to include the % identity to TetM found in Mycoplasma hominis and Ureaplasma
13. L315-317. Please italicize the taxa names
14. L311 Is Rpp the standard name for a new Tet determinant?
15. L354 section. It would be useful to comment on how many of the three rrl genes in fully sequenced bovirhinis isolates have been found to be mutated. Can the melt curves distinguish 1 vs 2 mutant copies?
16. L374 Please add a reference for this fact; also L453 after "effectiveness"
17. L392 In the discussion the tet pprotein matches are reported to be 60-70% but in the results they are 77-98%. Is one reporting nucleotide and the other amino acids? Please clarify in the text.
18. L503 Please give a source or reference for Hayflick-please note that it is usually Hayflick rather than Hayflick's
19. L509 Please use italics for de novo
20. L537 Does the pMD20 vector come with the kit? If not please give reference or source. Also, if it has the overhang for T cloning, how was the empty vector generated as a control?
21. L595 Mollicutes (capitalized)
22. L599 subsp. capricolum (spelling)
23. Were all the type strain DNAs already available? Can F38 be grown outside of high level biocontainment?
24. Table 1. I found the MIC for JM109 and spectinomycin to be rather high. Typically 100 ug/mL is used for selection of resistance and JM109 does not appear to have an S5 mutation in its genotype.
25. Even if only briefly with a few overview comments (rather than being an exhaustive analysis/commentary), it would be interesting to know if the resistant isolates to antimicrobial X were all geographically clustered, or widely distributed in Japan. Also, are trends in emergence and increased numbers of resistant isolates observed over the 1996-2023 window? There might not be enough for statistical significance, but would be interesting as a potential trend.

Reviewer #2 (Comments for the Author):

The data presented in this paper are unique, as prophage-related regions carrying aminoglycoside-modifying enzymes and RPP have not previously been identified in Mycoplasma spp (except for the previous publication of the author). Furthermore, the authors demonstrate that this occurrence is relatively common rather than rare.

My concerns:
Introduction
M. bovirhinis is known to be a commensal bacterium (as also mentioned by the author in line 44). It would be useful to clarify whether there is any policy regarding antimicrobial treatment of this mycoplasma in Japan.
L:50-52, 72-75, 75-76.
Although the manuscript discusses broad phenomena-such as the emergence of field isolates with decreased susceptibility to antimicrobials and the development of genetic methods for determining antimicrobial susceptibility-the supporting references remain relatively narrow and are predominantly derived from the author's own studies This limited citation scope does not adequately reflect the wider scientific literature on the topic and may give an incomplete view of the current state of research. Incorporating findings from multiple independent groups would better situate the work within the broader scientific context.

L:68; 76
In addition, several recently published papers resulting from joint consortium efforts by multiple groups specializing in this field would be important to cite alongside the work published by Hannan some time ago (2000).
doi: 10.3389/fvets.2025.1699077
doi: 10.1186/s12917-025-05154-4

Results
Antimicrobial susceptibility testing
L92-93 and 105-106.

From the data presented, it is not clear whether the distribution of flumequine MIC values is normal. The fact that the MIC value for PG43 is 2, while almost 90 strains show MIC values of {greater than or equal to}32, may suggest that these isolates are resistant to flumequine. Although no established breakpoints exist for flumequine, the statement that MIC value of PG43T was not as high (MICPG43T: 2 g/mL)" is susceptible appears somewhat speculative. In some Mycoplasma species, an MIC of {greater than or equal to}1 for enrofloxacin-a newer-generation fluoroquinolone-already indicates resistance.

Are there any 16S rRNA gene substitutions, known to increase susceptibility to spectinomycin, streptomycin, or tetracyclines in other Mycoplasma spp., identified in M. bovirhinis? If so, please mention this in Fig. 1.

Fig. 1
Please add description for superscripts "a and b" as well as for rpp.
If there is a G2057A mutation at 23S rRNA, which is known to be responsible for intrinsic resistance to erythromycin in other Mycoplasma spp., please mention it as a possible source for high MIC to erythromycin.

Genome analysis of M. bovirhinis HAZ2616"
Since there is no "genome analysis of M. bovirhinis HAZ2616", the subtitle should be revised accordingly to accurately reflect the data presented."
Including at least a paragraph comparing the two prophage regions-not limited to the antibiotic-related regions-would be a valuable addition to the manuscript and is strongly recommended.
L: 120-127: Please short this paragraph.
Since there is a difference in the length of aadE and sat4 between the two M. bovirhinis isolates, resulting in a truncated AadE in HAZ141_2 and a truncated Sat4 in HAZ_2616, could you specify the amino acid lengths and sequence homology of these proteins both between the two M. bovirhinis strains and between M. bovirhinis HAZ_2616 and other bacteria?
I noticed that this information is presented in Fig. S1; however, this figure is not referenced in the Results section describing AadE and Sat4. Fig. S1 is important, but the order of data presentation is somewhat inconsistent. The data for AadE and Sat4 appear in panels C and D and are not mentioned in the text, whereas the data for GyrA and ParC are shown in panels A and B and are referenced later in the Results section.
It will be worth adding a comparison of ParC and ParC' sequences.
I suggest re-titling FIG 2. Comparison of predicted insertion sequences between M. bovirhinis HAZ141_2 (GenBank accession no. AP018135) and M. bovirhinis HAZ2616 (GenBank accession no. AP038809) (schematic representation of the prophage-like genomic region).
Using "insertion sequences" (the connotation for IS elements) for prophage-like genomic region is a bit confusing.

Identifying genes and mutations associated with decreased susceptibility to
antimicrobial agents
This part presents some mix of the "results" and discussion, and should be fixed.

L202-210: The manuscript states that 'the numbers of isolates with missense mutations in GyrA and ParC and/or ParC′ were 98 and 34, respectively.' However, in the subsequent text only the 34 isolates are discussed. Could the authors clarify what the 98 isolates represent?

L250-259: These data have been previously published (doi: 10.1128/AAC.01010-20) and should be appropriately referenced and incorporated into the manuscript.

L267-269: E. coli JM109 used in this study has an unusually high MIC value of 128 mg/L to Spm (Table 1)?

L281-283 is a repetition of L279-281.

Rapid detection of genes and mutations associated with decreased susceptibility to
antimicrobial agents.
L351-353: I suggest omitting the use of the term 'susceptible' for strains carrying mutations solely in either GyrA or ParC. Additional data from other geographical locations should be accumulated to better understand the impact of individual GyrA or ParC mutations on resistance of M. bovirhinis; see also doi: 10.1111/j.1439-0450.2004.00748.x.)

Discussion
In my opinion, the Discussion does not adequately emphasize the novelty of the findings presented in this paper; it largely reiterates the Results. Some of the data currently mentioned in the Discussion would be more appropriately placed in the Results section.

The data presented in this paper are unique, as prophage-related regions carrying aminoglycoside-modifying enzymes and RPP have not previously been identified in Mycoplasma spp (except for the previous publication of the author). Furthermore, the authors demonstrate that this occurrence is relatively common rather than rare.

What is the earliest strain in which this prophage has been identified? Could the emergence of this prophage be linked to the extensive use of aminoglycosides in Japan?

Since M. bovirhinis is a commensal in the bovine upper respiratory tract, which is also a niche for pathogenic species such as M. bovis and M. alkalescens, is there any evidence for the presence of similar prophages in these or other Mycoplasma spp., as reported in Japan?
The presence of parC' region is also worth attention. What is a possible origin/function of parC' '? Is it functional at all. You mentioned L344-347 that primers for the parC' region amplified not only the target region but also a partial region of gyrA encoded in M. verecundum strain. Discuss it.

As I mentioned in the Introduction, M. bovirhinis is known to be a commensal bacterium. What is the practical, field-level usefulness of tracking the antimicrobial susceptibility of this strain, especially considering that cloning of the strains, at least for aphA-3, aadE, prr is needed?
The manuscript heavily cites the authors' prior publications, but it largely neglects a significant amount of relevant research conducted by other groups. That should be fixed.

Reviewer #3 (Public repository details (Required)):

The complete genome sequences need to be submitted to Genbank.

Reviewer #3 (Comments for the Author):

The manuscript 'Identification of genes and mutations involved in antimicrobial low susceptibility in Mycoplasma bovirhinis isolates from Japan and development of a genetic method for antimicrobial susceptibility discrimination' utilized 100 isolates of M. bovirhinis from cattle in Japan to determine susceptibility to antimicrobials and identify gene variations of the M. bovirhinis sequence. Overall, the data are of importance to the field as M. bovirhinis is associated with respiratory disease and this research may impact how cattle are managed. Specific points that resulted in this conclusion are listed below.

Abstract: Methods of how the results were obtained are not presented in the abstract. For example, need a sentence to state how the genes within the M. bovirhinis sequence were obtained. There also is no clear objective of the study stated at the beginning of the abstract. Why is this research being done?

Line 88: MIC needs to be defined when it is first used in the text.

Line 89: MIC50 and MIC90 values are presented in the results but not defined or mentioned in the materials and methods. What are MIC50 and MIC90? It is difficult to interpret the results in Lines 88-106 when MIC is not defined.

Line 90: A second closed parenthesis is needed. For example, (MICPG4390 T) (a quality control reference isolate) or (MICPG4390 T (a quality control reference isolate)).

Line 609: Assume there is an error in the time of the extension temperature. Currently it states 0s.

Figure 2: Why is some of the text in the figure red versus black? For example, rpp, aadE, sat4..... Is this because red indicates 100% identity between the two strains? This need to be specified in the figure legend.

Lines 372-373: The authors state that a specific gene cluster is widely detected in field strains. Overall, how prevalent are the different modified genes that are reported by the authors? For example, in a herd of 10,000 cattle, how many different variants would one expect to see? The authors state that one aim is to establish testing targeting these variants. Would an entire herd need to be tested to identify the predominant variants or just a small population of the herd?

The manuscript 'Identification of genes and mutations involved in antimicrobial low susceptibility in Mycoplasma bovirhinis isolates from Japan and development of a genetic method for antimicrobial susceptibility discrimination' utilized 100 isolates of M. bovirhinis from cattle in Japan to determine susceptibility to antimicrobials and identify gene variations of the M. bovirhinis sequence. Overall, the data are of importance to the field as M. bovirhinis is associated with respiratory disease and this research may impact how cattle are managed. Specific points that resulted in this conclusion are listed below.

Abstract: Methods of how the results were obtained are not presented in the abstract. For example, need a sentence to state how the genes within the M. bovirhinis sequence were obtained. There also is no clear objective of the study stated at the beginning of the abstract. Why is this research being done?

Line 88: MIC needs to be defined when it is first used in the text.

Line 89: MIC50 and MIC90 values are presented in the results but not defined or mentioned in the materials and methods. What are MIC50 and MIC90? It is difficult to interpret the results in Lines 88-106 when MIC is not defined.

Line 90: A second closed parenthesis is needed. For example, (MIC_{PG4390} ^{T}) (a quality control reference isolate) or (MIC_{PG4390} ^{T} (a quality control reference isolate)).

Line 609: Assume there is an error in the time of the extension temperature. Currently it states 0s.

Figure 2: Why is some of the text in the figure red versus black? For example, rpp, aadE, sat4….. Is this because red indicates 100% identity between the two strains? This need to be specified in the figure legend.

Lines 372-373: The authors state that a specific gene cluster is widely detected in field strains. Overall, how prevalent are the different modified genes that are reported by the authors? For example, in a herd of 10,000 cattle, how many different variants would one expect to see? The authors state that one aim is to establish testing targeting these variants. Would an entire herd need to be tested to identify the predominant variants or just a small population of the herd?

:Dear. Dr. Rogovsky and reviewers

Re: Spectrum00607-26 (Identification of genes and mutations involved in antimicrobial low susceptibility in Mycoplasma bovirhinis isolates from Japan and development of a genetic method for antimicrobial susceptibility discrimination)

Thank you for your valuable comments on this manuscript. We are grateful for your careful consideration. We have revised the manuscript in accordance with the reviewer's comments. Please find our detailed responses below.

Reviewer #1

1. L11 Perhaps add "antimicrobial" to the running head

   A: We apologize that this was not revised because doing so would have exceeded the word limit (not to exceed 54 characters and spaces).

2. L20 and L44 At least on first mention in each section, please add the new name.

   A: L20 and L41, Based on the comments, I added the new name enclosed in parentheses.

3. L51, L55, L63, L65, L415, L439, L451 and elsewhere if necessary. Please italicize "mycoplasma"

   A: L51, L55, L66, L68, L457, L477, L484, L487, and L519, I corrected the italicization of "mycoplasma".

4. L62 Perhaps use "should lead to the "

   A: L65, The correction was made based on the comments.

5. L66 takes 2 or more days.

   A: L69, The correction was made based on the comments.

6. L78 methods is important

   A: L80, The correction was made based on the comments.

7. L88 Please define MIC on first mention

   A: L94, For first mention, both the official name and its abbreviation are listed.

8. L155 to certain antimicrobial agents

   A: L161, I added "certain" based on the comments.

9. L309 helix 31 of the 16S rRNA component of the 30S subunit

   A: L337, Based on the comments, "helix 31 in the 30S subunit of the ribosome" was changed to "helix 31 of the 16S rRNA component of the 30S subunit of the ribosome."

10. L313 What does the size range here mean? The extent of homologous regions or different sizes of the various Tet family proteins?

    A: L342, This shows the number of amino acids in Rpp of HAZ2616 that match the amino acid sequences of TetM/TetW/TetO/TetS family proteins (639 aa in total length) reported in other bacterial species exemplified below.

    For example, 491 aa/ 639aa = 76.8% ≒ 77%. 624 aa/ 639 aa = 97.6% ≒ 98%

11. L313 Please specifically state that these are the highest identity matches. Plesae also see pt 17 below.

    A: L347-349, I added next sentence. "Incidentally, TetM/TetW/TetO/TetS family proteins from above-mentioned other bacterial species exhibit the highest amino acid sequence identity to the Rpp of strain HAZ2616."

12. L313 I think it would be useful to include the % identity to TetM found in Mycoplasma hominis and Ureaplasma.

    A: L349-355, L433-436, Fig S1E, Thank you for this important suggestion. We have now added a comparative analysis of the amino acid sequences of TetM from *Mycoplasma hominis* and *Ureaplasma urealyticum* to the Results section, Discussion section, and Figure S1.

13. L315-317. Please italicize the taxa names.

    A: L342-346, Thank you for pointing these out. I have corrected the errors accordingly.

14. L311 Is Rpp the standard name for a new Tet determinant ?

    A: Rpp is not the standard name for Tet determinants. However, the formal description would be "TetM/TetW/TetO/TetS family proteins," which is a very long description. I thought it was inappropriate to repeat the name, so I have written it as ribosomal protection protein (Rpp). If you think this is inappropriate, I will correct it. I would appreciate it if you could suggest a correction.

15. L354 section. It would be useful to comment on how many of the three *rrl* genes in fully sequenced bovirhinis isolates have been found to be mutated. Can the melt curves distinguish 1 vs 2 mutant copies ?

    A: We have also carefully considered this point and evaluated the trend in *M. bovis*. Although not mentioned in Appl Environ Microbiol. 85:e00575-19, our analysis showed that no significant difference in MIC changes was observed between strains in which all *rrs* or *rrl* targets were mutated and those in which only a subset of targets carried mutations. A similar tendency was observed in *M. bovirhinis*, and this information has been added to L203–210 of the revised manuscript. In addition, accurately distinguishing between one versus two mutant copies is technically challenging when using melting curve analysis.

16. L374 Please add a reference for this fact; also L453 after "effectiveness"

    A: L412, L489, I have added the references.

17. L392 In the discussion the tet protein matches are reported to be 60-70% but in the results they are 77-98%. Is one reporting nucleotide and the other amino acids? Please clarify in the text.

    A: Thank you for pointing that out. It was a mistake on my part. I have omitted this sentence (L433-436).

18. L503 Please give a source or reference for Hayflick-please note that it is usually Hayflick rather than Hayflick's.

    A: Thank you for pointing these out. I corrected the spelling of "Hayflick's" to "Hayflick". (L547, L607) Moreover, I have added the reference. (L547, Reference no. 29)

19. L509 Please use italics for de novo

    A: L553, I have revised it according to your suggestion.

20. L537 Does the pMD20 vector come with the kit? If not please give reference or source. Also, if it has the overhang for T cloning, how was the empty vector generated as a control ?

    A: The pMD20 vector is included in the kit. However, the sequencing information of this vector is not registered with NCBI. Instead, it is available on Takara Bio's Japanese website. (https://catalog.takara-bio.co.jp/product/basic_info.php?unitid=U100006304)

    This vector allows for blue–white selection; therefore, following transformation into *E. coli* JM109, all blue colonies contained empty vectors. These blue colonies were subjected to PCR

amplification using the M13 primer RV and M13 primer M4, followed by DNA sequencing to confirm that no insert was present in the insertion region (L595). These blue colonies were used as controls in antimicrobial susceptibility testing.

21. L595 Mollicutes (capitalized)
    A: L639, I have revised it according to your suggestion.

22. L599 subsp. capricolum (spelling)
    A: L643, Thank you for pointing out the spelling mistake. I have revised it according to your suggestion.

23. Were all the type strain DNAs already available? Can F38 be grown outside of high level biocontainment ?
    A: As you pointed out, F38 is difficult to culture in commonly used mycoplasma culture media (e.g., Hayflick's medium and BHL). We believe that the use of CCPP medium or a similar medium (https://www.woah.org/fileadmin/Home/eng/Health_standards/tahm/3.08.04_CCPP.pdf) is essential. In this study, a pre-prepared DNA solution was used. Whether the DNA sample was suitable for use as a template for PCR amplification was confirmed by PCR using primers targeting the 16S ribosomal RNA gene (J Microbiol Methods.1992;16 271-279). In addition, the bacterial strain was verified by DNA sequencing of the same PCR product.

24. Table 1. I found the MIC for JM109 and spectinomycin to be rather high. Typically 100 ug/mL is used for selection of resistance and JM109 does not appear to have an S5 mutation in its genotype.
    A: I had the same concern regarding that point, namely that the MIC of JM109 for spectinomycin might be too high. I then prepared a new culture medium and fresh antimicrobial solutions and repeated the antimicrobial susceptibility test, but obtained the same results. This suggests good reproducibility; would it be appropriate to publish these data?

25. Even if only briefly with a few overview comments (rather than being an exhaustive analysis/commentary), it would be interesting to know if the resistant isolates to antimicrobial X were all geographically clustered, or widely distributed in Japan. Also, are trends in emergence and increased numbers of resistant isolates observed over the 1996-2023 window? There might

not be enough for statistical significance, but would be interesting as a potential trend.

A: Thank you for your valuable comments. I have added detailed information on various genes and mutations associated with decreased antimicrobial susceptibility, as well as the isolation periods and geographic distributions of field strains harboring these determinants (L210–213, L232–235, L327–333, and L365–367). Although temporal or geographic clustering of decreased-susceptibility strains could not be determined from the available data, we have described that such low-susceptibility strains are already widespread throughout the country (L412–416, L459-460, L484-486).

Reviewer #2

1. Introduction, *M. bovirhinis* is known to be a commensal bacterium (as also mentioned by the author in line 44). It would be useful to clarify whether there is any policy regarding antimicrobial treatment of this mycoplasma in Japan.
   A: L43-46, Thank you for your valuable suggestion. I have added the sentence as suggested. While there are studies on antibiotic treatment for respiratory diseases (Can J Vet Res. 1992 Jul;56(3):177-83.), knowledge regarding antibiotic therapy specifically targeting *M. bovirhinis* remains limited. Nevertheless, a decreasing in the overall bacterial load (*Pasteurella* spp., *Histophilus somni*) has been reported. (In this report, the antibacterial agents used were penicillin and trimethoprimsulfadoxine; therefore, their activity against mycoplasma is not relevant.) It would be highly informative to investigate how the incidence of pneumonia and subsequent pathological findings change when the diagnostic techniques described in this study are applied and antibiotics effective against *M. bovirhinis* are administered.

2. L:50-52, 72-75, 75-76. Although the manuscript discusses broad phenomena-such as the emergence of field isolates with decreased susceptibility to antimicrobials and the development of genetic methods for determining antimicrobial susceptibility-the supporting references remain relatively narrow and are predominantly derived from the author's own studies This limited citation scope does not adequately reflect the wider scientific literature on the topic and may give an incomplete view of the current state of research. Incorporating findings from multiple independent groups would better situate the work within the broader scientific context.
   L:68; 76, In addition, several recently published papers resulting from joint consortium efforts by multiple groups specializing in this field would be important to cite alongside the work published by Hannan some time ago (2000).
   doi: 10.3389/fvets.2025.1699077
   doi: 10.1186/s12917-025-05154-4
   A: I agree that this is very reasonable advice. Thank you also for providing helpful scientific report. These findings have been incorporated into this paper. I cited the former as a valuable and up-to-date review of genetic antimicrobial susceptibility determination techniques for mycoplasma in veterinary medicine (Reference no.13). This review article is extremely useful in explaining the significance of this paper. The latter provided valuable insights into the shortcomings of conventional methods for drug susceptibility testing of mycoplasma in veterinary medicine, namely, standardization of methods, interpretation of results, and setting of

breakpoints (Reference no.15).

3. Results Antimicrobial susceptibility testing L92-93 and L105-106. From the data presented, it is not clear whether the distribution of flumequine MIC values is normal. The fact that the MIC value for PG43 is 2, while almost 90 strains show MIC values of {greater than or equal to}32, may suggest that these isolates are resistant to flumequine. Although no established breakpoints exist for flumequine, the statement that MIC value of PG43T was not as high ($MIC_{PG43}{}^{T}$: 2 µg/mL)" is susceptible appears somewhat speculative. In some Mycoplasma species, an MIC of {greater than or equal to}1 for enrofloxacin-a newer-generation fluoroquinolone-already indicates resistance.

   A: L111-112, Thank you for your advice. I have removed "···, but the MIC value of $PG43^T$ was not as high (MIC_{PG43^T}: 2 µg/mL)" as you suggested.

4. Are there any 16S rRNA gene substitutions, known to increase susceptibility to spectinomycin, streptomycin, or tetracyclines in other Mycoplasma spp., identified in *M. bovirhinis*? If so, please mention this in Fig. 1.

   A: No mutations were found in *rrs*A965, *rrs*A967, or *rrs*C1192, which have been reported to be associated with decreased susceptibility to tetracycline or spectinomycin in other *Mycoplasma* species. These findings are described in the main text (L236-238, L292-296, L334-339), and the legend in Figure 1 (L812-815) have been added in response to the comments. The mutation *rrs*C912T (*E. coli* numbering), which has been reported in *M. capricolum* subsp. *capripneumoniae* to be associated with streptomycin hyposensitivity, was detected in all field strains of *M. bovirhinis*, including $PG43^T$, irrespective of whether streptomycin susceptibility was decreased. Therefore, this mutation is presumed to be unrelated to streptomycin hyposensitivity in *M. bovirhinis*. I have added this result to the main text (L292-296); however, I concluded that no additional information was required in the legend for Figure 1.

5. Fig. 1 Please add description for superscripts "a and b" as well as for *rpp*.

   A: L810-819, I added description for superscripts "a and b" and all abbreviations.

6. If there is a G2057A mutation at 23S rRNA, which is known to be responsible for intrinsic resistance to erythromycin in other Mycoplasma spp., please mention it as a possible source for high MIC to erythromycin.

   A: L808-810, I have corrected the sentence as suggested.

7. Genome analysis of M. bovirhinis HAZ2616" Since there is no "genome analysis of *M. bovirhinis* HAZ2616", the subtitle should be revised accordingly to accurately reflect the data presented."

   A: L114-115, I have revised the subtitle to better reflect the results.

8. Including at least a paragraph comparing the two prophage regions-not limited to the antibiotic-related regions-would be a valuable addition to the manuscript and is strongly recommended.

   A: L122-181, We have already examined the prophage regions and QRDRs of HAZ2616 and HAZ141_2 in detail, and no additional information is available. Is it acceptable to proceed as it is?

9. L: 120-127: Please short this paragraph.

   A: L128-132, I have revised the text to concisely summarize the paragraph you raised.

10. Since there is a difference in the length of *aadE* and *sat4* between the two *M. bovirhinis* isolates, resulting in a truncated AadE in HAZ141_2 and a truncated Sat4 in HAZ_2616, could you specify the amino acid lengths and sequence homology of these proteins both between the two *M. bovirhinis* strains and between *M. bovirhinis* HAZ_2616 and other bacteria?

    I noticed that this information is presented in Fig. S1; however, this figure is not referenced in the Results section describing AadE and Sat4. Fig. S1 is important, but the order of data presentation is somewhat inconsistent. The data for AadE and Sat4 appear in panels C and D and are not mentioned in the text, whereas the data for GyrA and ParC are shown in panels A and B and are referenced later in the Results section.

    A: The panels in Fig S1 have been reordered to match their order of appearance in the text. AadE (Fig. S1C) is discussed in the Results section (L263-278), while Sat4 (Fig. S1D) is discussed in both the Results and Discussion sections (L297-306, L310-312, L448-456).

11. It will be worth adding a comparison of ParC and ParC' sequences.

    A: In response to the point you raised, Fig S1A presented the results of a comparative analysis of the amino acid sequences of ParC from three strains with publicly available genome data (i.e. PG43^{T}, GS01, and HAZ141_2), as well as ParC′ from strain HAZ2616.

12. I suggest re-titling FIG 2. Comparison of predicted insertion sequences between *M. bovirhinis*

HAZ141_2 (GenBank accession no. AP018135) and *M. bovirhinis* HAZ2616 (GenBank accession no. AP038809) (schematic representation of the prophage-like genomic region). Using "insertion sequences" (the connotation for IS elements) for prophage-like genomic region is a bit confusing

A: L821, Following your suggestion, I have replaced “predicted insertion sequences” with “a prophage-like genomic region.”

13. Identifying genes and mutations associated with decreased susceptibility to antimicrobial agents. This part presents some mix of the "results" and discussion, and should be fixed.

    A: When identifying factors associated with decreased antimicrobial susceptibility, the analysis is necessarily based on previously published knowledge and therefore requires appropriate citation of the relevant literature. If neither the underlying knowledge nor the corresponding references are provided, readers may find it difficult to understand the rationale for focusing on each factor. I believe that the present manuscript more clearly explains the process by which factors contributing to reduced drug susceptibility were identified. Therefore, although this section is part of the Results, it is written in a style more typical of the Discussion. I would appreciate it if you could offer any advice on how to revise this section.

14. L202-210: The manuscript states that 'the numbers of isolates with missense mutations in GyrA and ParC and/or ParC′ were 98 and 34, respectively.' However, in the subsequent text only the 34 isolates are discussed. Could the authors clarify what the 98 isolates represent?

    A: L219-223, A missense mutation in GyrA was identified in 98 of 100 strains, suggesting that most *M. bovirhinis* field strains possess this mutation. Missense mutations in ParC and/or ParC′ were observed in 34 strains, all of which also carried a GyrA missense mutation. I have revised this section of the text.

15. L250-259: These data have been previously published (doi: 10.1128/AAC.01010-20) and should be appropriately referenced and incorporated into the manuscript.

    A: L278, I have added the references as requested.

16. L281-283 is a repetition of L279-281.

    A: Thank you for pointing this out. The repetitive sentence has been removed.

17. Rapid detection of genes and mutations associated with decreased susceptibility to

antimicrobial agents.

L351-353: I suggest omitting the use of the term 'susceptible' for strains carrying mutations solely in either GyrA or ParC. Additional data from other geographical locations should be accumulated to better understand the impact of individual GyrA or ParC mutations on resistance of M. bovirhinis; see also doi: 10.1111/j.1439-0450.2004.00748.x.)

A: L389-391, I agree that the term “susceptible strain” is inappropriate, and I have revised this part of the text.

18. Discussion

In my opinion, the Discussion does not adequately emphasize the novelty of the findings presented in this paper; it largely reiterates the Results. Some of the data currently mentioned in the Discussion would be more appropriately placed in the Results section.

The data presented in this paper are unique, as prophage-related regions carrying aminoglycoside-modifying enzymes and RPP have not previously been identified in Mycoplasma *spp* (except for the previous publication of the author). Furthermore, the authors demonstrate that this occurrence is relatively common rather than rare. What is the earliest strain in which this prophage has been identified? Could the emergence of this prophage be linked to the extensive use of aminoglycosides in Japan?

A: L327-333, L365-367, I have added two sentences in response to your question Unfortunately, I do not have data linking the use of aminoglycoside preparations to the emergence of low-susceptible M. bovirhinis strains; therefore, no definitive conclusion can be drawn. Accordingly, I would like to refrain from commenting on this point in this article.

19. Since *M. bovirhinis* is a commensal in the bovine upper respiratory tract, which is also a niche for pathogenic species such as *M. bovis* and *M. alkalescens*, is there any evidence for the presence of similar prophages in these or other Mycoplasma spp., as reported in Japan?

A: At present, no similar prophage regions have been identified in species other than *M. bovirhinis*. However, this is an interesting topic, and I want to investigate the prevalence of this prophage region in future studies.

20. The presence of *parC*' region is also worth attention. What is a possible origin/function of *parC'* '? Is it functional at all. You mentioned L344-347 that primers for the *parC*' region amplified not only the target region but also a partial region of *gyrA* encoded in *M. verecundum* strain. Discuss it.

A: The amino acid sequence of ParC′ from HAZ2616 exhibited a high degree of similarity (99%, 841/853 aa) to ParC from Mycoplasma sp. 6934 (NCBI Reference Sequence: WP_465101120.1). This finding strongly suggests that ParC from this species may represent the origin of ParC′ from *M. bovirhinis*; however, the reverse scenario is also possible—that is, ParC′ from *M. bovirhinis* may be the origin of ParC from Mycoplasma sp. 6934.

GyrA, GyrB, ParC, and ParE are essential enzymes involved in DNA synthesis, and it is generally believed that loss or impairment of any of these functions would compromise cellular survival. Because three *M. bovirhinis* field strains in Japan have been confirmed to carry *parC′* but not *parC*, it is highly likely that *parC′* is functional (L174-177).

The cause of the nonspecific amplification of *gyrA* in *M. verecundum* 107^T during PCR with *parC′* detection primers was investigated and discussed (L504-510).

21. As I mentioned in the Introduction, *M. bovirhinis* is known to be a commensal bacterium. What is the practical, field-level usefulness of tracking the antimicrobial susceptibility of this strain, especially considering that cloning of the strains, at least for *aphA-3*, *aadE*, *rpp* is needed?

A: L43-46, L519-521, Although the symbiotic nature of mycoplasmas has long been recognized, concerns have been raised regarding their potential to suppress immune function and promote infection by pathogenic bacteria. In some cases of bovine mycoplasma infection, the use of appropriately selected antimicrobial agents has led to marked bacterial elimination and alleviation of clinical symptoms. However, the extent to which antimicrobial agents are effective in reducing symptoms in *Mycoplasma bovirhinis* infections remains poorly understood. It is possible that the introduction of this approach into treatment could yield favorable outcomes, a point that was strongly emphasized in this study.

22. The manuscript heavily cites the authors' prior publications, but it largely neglects a significant amount of relevant research conducted by other groups. That should be fixed.

A: We have focused our research on the antimicrobial susceptibility of bovine mycoplasmas and have submitted related papers that form the basis of this study. However, these findings are not solely derived from our own work; rather, they are based on reports from numerous researchers. We believe that our conclusions do not differ substantially from those reported by other investigators. As suggested in point 2, we would appreciate any recommendations regarding literature that could be helpful in improving the content of this paper.

Reviewer #3

1. The complete genome sequences need to be submitted to Genbank.

   A: The complete genome sequence of HAZ2616 has already been published. Please check the following URL.

   https://www.ncbi.nlm.nih.gov/nuccore/AP038809

   https://www.ncbi.nlm.nih.gov/nuccore/AP038809.1?report=fasta

2. Abstract: Methods of how the results were obtained are not presented in the abstract. For example, need a sentence to state how the genes within the *M. bovirhinis* sequence were obtained. There also is no clear objective of the study stated at the beginning of the abstract. Why is this research being done?

   A: L21-25, A brief introductory explanation of the study's purpose and methods was added at the beginning.

3. Line 88: MIC needs to be defined when it is first used in the text.

   A: L94, Thank you for your comment. We have revised the manuscript accordingly.

4. Line 89: MIC50 and MIC90 values are presented in the results but not defined or mentioned in the materials and methods. What are MIC50 and MIC90? It is difficult to interpret the results in Lines 88-106 when MIC is not defined.

   A: L94-98, We have added descriptions explaining the MIC_{50} and MIC_{90} values.

5. Line 90: A second closed parenthesis is needed. For example, (MICPG4390 T) (a quality control reference isolate) or (MICPG4390 T (a quality control reference isolate)).

   A: L97-98, Thank you for your comment. We overlooked this point and have corrected it as suggested.

6. Line 609: Assume there is an error in the time of the extension temperature. Currently it states 0s.

   A: When entering the melting-curve reaction program into the LightCycler system, we set the final reaction time at 70 °C to 0 s. This setting is intentional and not an error. Please refer to our previous publication for examples. (Appl Environ Microbiol. 2019 Jun 17;85(13):e00575-19.)

7. Figure 2: Why is some of the text in the figure red versus black? For example, rpp, aadE, sat4.....

Is this because red indicates 100% identity between the two strains? This need to be specified in the figure legend.

A: L827-828, Thank you for your comment. We overlooked this point and have added an explanation accordingly.

8. Lines 372-373: The authors state that a specific gene cluster is widely detected in field strains. Overall, how prevalent are the different modified genes that are reported by the authors? For example, in a herd of 10,000 cattle, how many different variants would one expect to see? The authors state that one aim is to establish testing targeting these variants. Would an entire herd need to be tested to identify the predominant variants or just a small population of the herd?

   A: First, I would like to state that because this survey was conducted exclusively in Japan, it is difficult to infer the situation in other countries. With the exception of the *rrl* A2058 mutant, field strains carrying the genes and mutations associated with decreased antimicrobial susceptibility identified in this study are estimated to be widely distributed across almost all prefectures by 2026. This estimation is based on the fact that these genes and mutations have already been detected for approximately 20 years. In Japan, cattle movement between regions is extensive, and it is well documented that when a field strain of *M. bovis* with a specific MLST type is first identified in Japan, it typically spreads across large areas of the country within approximately five years; over time, such dominant strains are subsequently replaced. (Jpn Agric Res Q 2023;57 111–122. Fig 2) It is likely that a similar situation also applies to *M. bovirhinis*. You asked how many field strains harboring these genes or mutations might be detected in a herd of 10,000 cattle; however, such an estimate cannot be made based solely on these data. To accurately understand the extent to which these genes and mutant wild strains have spread within each cattle herd, I believe it is necessary to analyze as many samples as possible. However, testing all adult cattle would not be practical from a labor standpoint. In cases of *M. bovis* infection, the infection status of calf herds reflected that of the corresponding adult cattle herds. Farming practices and hygiene management at each farm are, of course, likely to have a substantial influence on the testing results. From a labor perspective, particularly with regard to the ease of sample collection, collecting as many samples as possible from calves appears to be the most efficient testing strategy.

Re: Spectrum00607-26R1 (Identification of genes and mutations involved in antimicrobial low susceptibility in *Mycoplasma bovirhinis* isolates from Japan and development of a genetic method for antimicrobial susceptibility discrimination)

Dear Dr. Eiji Hata:

Your manuscript has been accepted, and I am forwarding it to the ASM production staff for publication. Your paper will first be checked to make sure all elements meet the technical requirements. ASM staff will contact you if anything needs to be revised before copyediting and production can begin. Otherwise, you will be notified when your proofs are ready to be viewed.

Data Availability: ASM policy requires that data be available to the public upon online posting of the article, so please verify all links to sequence records, if present, and make sure that each number retrieves the full record of the data. If a new accession number is not linked or a link is broken, provide production staff with the correct URL for the record. If the accession numbers for new data are not publicly accessible before the expected online posting of the article, publication may be delayed; please contact ASM production staff immediately with the expected release date.

Publication Fees: For information on publication fees and which article types have charges, please visit our website. We have partnered with Copyright Clearance Center (CCC) to collect author charges. If fees apply to your paper, you will receive a message from no-reply@copyright.com with further instructions. For questions related to paying charges through RightsLink, please contact CCC at ASM_Support@copyright.com or toll free at +1-877-622-5543. CCC makes every attempt to respond to all emails within 24 hours.

ASM Membership: Corresponding authors may join or renew ASM membership to obtain discounts on publication fees. Need to upgrade your membership level? Please contact Customer Service at Service@asmusa.org.

Embargo Policy: A press release may be issued as soon as the manuscript is posted on the Spectrum Latest Articles webpage. The corresponding author will receive an email with the subject line "ASM Journals Author Services Notification" when the article is available online.

The ASM Journals program strives for constant improvement in our submission and publication process. Please tell us how we can improve your experience by taking this quick Author Survey.

Thank you for submitting your paper to Spectrum.

Sincerely,
Artem Rogovsky
Editor
Microbiology Spectrum
